# How do patients value and prioritize patient portal functionalities and usage factors? A conjoint analysis study with chronically ill patients

**DOI:** 10.1186/s12911-018-0708-5

**Published:** 2018-11-21

**Authors:** Gaby Anne Wildenbos, Frank Horenberg, Monique Jaspers, Linda Peute, Danielle Sent

**Affiliations:** 1 Center for Human Factors Engineering of Health Information Technology, Department of Medical Informatics, PO Box 22660, 1100 DD Amsterdam, The Netherlands; 2 Department of Medical Informatics, Amsterdam Public Health research institute, Amsterdam UMC, University of Amsterdam, PO Box 22660, 1100 Meibergdreef 9 , DD Amsterdam, The Netherlands; 3 ZIVVER, PO Box 75293, 1070 AG Amsterdam, The Netherlands

**Keywords:** Conjoint analysis, Patient portal, Elderly, Patient preferences, Technology acceptance

## Abstract

**Background:**

Patient portal use can be a stimulant for patient engagement. Yet, the heterogeneous landscape of tethered patient portals, is a major barrier to further portal development and implementation. A variety in portal access means, functionalities, usability and usefulness exists; without having accurate sight on patient perspectives. We aimed to get insights on possible coherence between patients’ preferred usage factors of portals and patients’ prioritization of functionalities, within the complexity of their disease management across different healthcare organizations.

**Methods:**

A conjoint analysis questionnaire was sent to patient panels of two large patient associations in The Netherlands, centered on heart and vascular diseases and lung diseases.

**Results:**

Of 1294 patient respondents, 81% were 55+ years old and 49% were 65+ years old. Overall respondents significantly prioritized user-friendly access to a portal, via a laptop or desktop. Patients aged < 65 were less negative about using tablets to access a portal compared to the total respondents. Patients had no preference for a digital interoperable export functionality; most respondents preferred to create printable overviews. Built-in publication delay of two weeks for medical information was not preferred. Our results show no significant preference of patients between ‘instant publication’ versus ‘publication after new information has been explained by a healthcare provider’. Overall respondents and experienced portal users had a strong preference to be able to communicate with their provider via a portal and to use a portal providing information from multiple providers. Lung patients preferred information from one provider and did not require the possibility to ask online questions.

**Conclusions:**

Heart and vascular patients as well as lung patients prefer similar technical patient portal aspects, independent of their medical condition. Yet, in current portals consistency on this matter is lacking. It is highly assumable that offering a more consistent user-experience across the variety of patient portals could help increase patient portal acceptance, ultimately helping to stimulate patient engagement via patient portal use. We further affirm the need for customization on medical information publication and sharing information of various providers through patient portals, where information provision can be adapted to preferences of patients related to their medical condition(s).

**Electronic supplementary material:**

The online version of this article (10.1186/s12911-018-0708-5) contains supplementary material, which is available to authorized users.

## Background

Patients nowadays live in an information driven world, where they can get support in self-management of their health and conditions by accessing their medical record and communicating with healthcare providers via patient portals [1]. Especially older patients, chronically ill patients and patients with co-morbidities can benefit from patient portal use [1–4]. Patients can be assisted in accomplishing health-related and administrative tasks by having access to personal health information, such as laboratory test results and appointment information, as well as digital opportunities for patient-provider communication. The foremost type of a patient portal is the tethered patient portal. A tethered patient portal is an application build on an Electronic Health Record (EHR) infrastructure of a specific healthcare organization [5]. This organization manages the portal and decides which information can be accessed by the patient. In this aspect, the tethered patient portal differs from a personal health record, in which the patient can collect health data and he/she decides whether to share that data with providers or family members. Most tethered patient portals hold medical information that is derived from the EHR, such as a discharge summary, a medication and allergy list and laboratory results [4, 6–8]. Portals can include more interactive features and allow patients to send secure messages to clinical staff, schedule appointments or request prescription refills [4].

Driven by innovation goals for eHealth set by the Dutch government, the tethered patient portal is now increasingly being implemented in The Netherlands [9]. Yet, due to a lack of a clear strategy and vision on portal functionalities, many different portals exist in the Dutch market; a market-scan identified 34 portals in 2015, each varying in provided functionalities and interface [9]. This heterogeneous landscape of patient portal products is a main barrier in development and implementation activities [9, 10]. Patient engagement through patient portal use is jeopardized, due to variable access and usability, creating user-friendliness problems for patients. Further, patients may have doubts regarding a portal’s usefulness, since there is no standard set of portal functionalities [10]. This heterogeneity further results in interoperability problems, complicating the exchange of information between portals of different healthcare organizations. Patient engagement and interoperability problems may pose particular issues to older chronically ill patients and patients with (multiple) diseases. These patients often receive care among a variety of healthcare centers and obtain medical information from these centers. In addition, these are often older patients, experiencing motivational barriers in using eHealth [2, 6]. In managing their care with the scattered information provided by different healthcare organizations, such barriers may prevent them to use patient portals.

A solution to these problems is to design more uniform portals, meeting the disease management needs of patients defined by their characteristics and associated preferences and capacities [10]. Previous research provides insights on patient preferences in relation to patient portal functionalities and use, such as the ability to view test results via a portal [4, 11], and recognized the (feasibility) patient portal usage by chronically ill patients, such as cardiovascular or lung disease patients [3, 4, 12–14]. There is nevertheless little evidence of how patient preferences on specific portal functionalities and usage factors are correlated to each other and how this is valued by patients within the context of the full portal product. Insights are further needed on how sharing of medical information through a portal, possible coherence of portal functionalities, and usage factors fit the complexity of disease management across different healthcare organizations. Our research project aimed to advance the understanding on this matter by using a conjoint analysis approach to examine how patients of the ‘Harteraad’ and the ‘Longfonds’ patient panels, two recognized Dutch patient associations respectively centered on cardiovascular diseases and lung diseases, value and prioritize specific portal functionalities and usage factors.

## Methods

### Study design: Conjoint analysis questionnaire

This study used a conjoint analysis questionnaire. ‘Conjoint analysis’ is a survey-based statistical technique mainly used in market research that helps determine how people value different attributes of a product [15, 16]. The objective of conjoint analysis is to determine what combination of a limited number of attributes is most influential on a respondent’s choice or decision making. We used a discrete choice experiment conjoint analysis in our study, since is the most common type of conjoint analysis used in health economics, outcomes research, and health services research [15]. This type of conjoint analysis consists of two steps: 1) a choice experiment with respondents and 2) a statistical analysis. In the choice experiment, the attributes are used to describe a certain product and can consist of one or more levels. Different fictive profiles, possible variations of the product, can be created by combining various attributes and levels, which are then shown to a respondent to determine which combination he/she prefers. For instance, a product can be a tablet that is described by various attributes and levels of those attributes: price (levels $100, $200), screen size (levels 8.9, 10.1) and battery length (levels 14 h, 9 h). Fictive profiles can be presented to a respondent, such as:

A) the tablet costs $100, has screen size 10.1 and a battery length of 9 h;
B) the tablet costs $100, has screen size 8.9 and a battery length of 14 h;
C) the tablet costs $200, has screen size 8.9 and a battery length of 14 h.

The respondent can choose option A, B or C in the trade-off process whether he/she would buy that tablet. This process is repeated for various fictional profiles. In the second step of the conjoint analysis, the statistical analysis of all respondents’ choices for all presented profiles, the relative importance of different attributes and the trade-offs between these attributes are statistically determined [15–17]. In other questionnaire based methods to measure respondents’ preferences, such as rating or interest questions, it is often not measurable to accurately value how much a certain attribute would influence a respondents’ choice in preferring one attribute above the other [18, 19]. However, choice-based conjoint analysis does determine which individual attributes and levels are favored over others. Conjoint analysis is gaining popularity in the health care setting [17], where it recently has been used to assess patients’ preferences regarding pharmacological treatment for bipolar depression [20] and to examine how older adults rate and identify the importance of healthcare seeking and utilization aspects in the United States [21].

### Study protocol

We conducted a comprehensive literature review (Additional files 1 and 2) in which 42 factors were initially found influencing the use of patient portals by patients. Based on this review, four experts in healthcare discussed recurrent as well as meaningful factors that could be used as an attribute and defined the attributes and levels to be used in the conjoint analysis. Seven attributes were constructed for our conjoint analysis, shown in Table 1, each consisting of three assigned levels. The fictional profiles of patient portals presented in our questionnaire were generated using the orthogonal main effects plan: instead of presenting all possible combinations (levels^attributes^ = 3^7^ = 2187), the smallest manageable combination of profiles to test with respondents are presented - knowing that the statistical analysis will be able to balance how often a specific level is presented to a respondent. By means of 18 profiles our respondents were asked to choose their preferences for patient portals. Additional questions were asked to gain sight on respondents’ demographic characteristics, health status and experience with patient portal use. A potential bias of the questionnaire could have been that respondents would not be able to envision a patient portal by the items or wordings chosen in the questionnaire. For this reason the questionnaire was validated by means of cognitive interviews with six people representative for the target group. During these interviews, eight unique problems were identified. All issues were addressed by changing several formulations and the visual design of the questionnaire. The questionnaire is shown in Additional file 3.

**Table 1:** Attributes and levels used in conjoint analysis

Attribute	Level
Accessibility	1. Portal can be accessed via a computer (laptop and/or desktop)
	2. Portal can be accessed via a tablet (for example iPad)
	3. Portal can be accessed via a smartphone (for example iPhone)
Login	1. Username and password (least secure)
	2. DigiD with SMS verification (secure)
	3. Username, password and SMS verification (secure)
Interoperability	1. No export of data
	2. Export of data to non-interoperable format (e.g. PDF)
	3. Export of data to interoperable format (e.g. Continuous Care Document)
Availability of information	1. Direct publication of information
	2. Information delayed until discussed with provider
	3. Available after 2 weeks independent if information has been discussed with provider
Content	1. Reports and basic information (e.g. medication overviews and allergies)
	2. Reports and basic information and professional summary
	3. Complete uncensored medical file
Number of providers	1. Contains information from one provider (e.g. hospital or general practitioner)
	2. Contains information from multiple providers (e.g. hospitals and general practitioner)
Patient-provider communication	1. No possibility to ask online questions
	2. Possibility to ask questions about medical data or previous visits in the portal
	3. Online in-patient consult

### Study population and data analysis

We recruited respondents by sending the questionnaire to patient panels of two Dutch patient associations: the Heart Council (HVG) and the Lung Fund (LF). Data was collected in the months April and May 2017 by means of the tool *spidox.net*. Members of the panels consisted of chronically ill patients with a cardiovascular disease and patients with a lung disease. Of the total chronically ill patient population in The Netherlands, 16% are cardiovascular patients and 11% are patients with a lung disease [22–24]. Members of the HVG and LF patient panels were therefore representative for the Dutch chronically ill population and all respondents were eligible for data analysis. Respondents were only excluded if 1) the questionnaire was not completed; 2) response time was under four minutes; 3) response time was above 60 min and 4) data entries were possible spam since they had the same IP address. To calculate the minimum required sample size of this study, we used the recommendations of Orme regarding sample size determination on choice experiment conjoint analysis [25]. Orme recommends a formula for this sample size determination: $\frac{n\ast t\ast a}{c}\le 500.$In this formula, n is the number of minimum respondents needed, t is the number of profiles presented to the respondents (18 in our study), a is the number of options to choose from per profile (3 in our study) and c is the highest level per attribute (3 in our study). The number 500 is the threshold for representing the main-effect level of interest in the statistical analysis, yet Orme explains this number is intended to be a minimum threshold. We chose to increase that threshold, in order to be certain of sufficient representations per main-effect level. We therefore used the following formula: $\frac{n\ast 18\ast 3}{3}\le 2000$. This resulted in a minimum required sample size of 111 respondents. Standard to conjoint analysis, a conditional logistic regression was used to analyze the data. The data analysis was performed using RStudio version 1.1.383, using packages support.CEs(0.4–1), survival (2.41–3), lmtest(0.9–35), plyr(1.8.4).

## Results

The questionnaire was sent to 3900 panel members; with a response rate of 34% this resulted in a total of 1307 respondents. After exclusion, 1294 respondents were included in the analysis. Table 2 shows the respondent characteristics per patient association. Most respondents are from the HVG (*n* = 929) and 81% of all respondents are 55 years old or above. More than half of the respondents proclaimed to currently have a good to excellent health status.

**Table 2:** Characteristics of respondents (*n* = 1.294)

	Overall *N* = 1.294	HVG *N* = 929	LF *N* = 365
Age (years)			
18–34	20 (1%)	6 (1%)	14 (4%)
35–44	49 (4%)	29 (3%)	20 (6%)
45–54	177 (14%)	121 (13%)	56 (15%)
55–64	416 (32%)	281 (30%)	135 (37%)
65–74	500 (39%)	388 (42%)	112 (31%)
> 75	132 (10%)	104 (11%)	28 (7%)
Gender			
Female (n) / Male (n)	549 (42%) / 745 (58%)	323 (35%) / 606 (65%)	226 (62%) / 139 (38%)
Educational level			
Low (primary/secondary)	276 (22%)	181 (20%)	69 (28%)
Intermediate (low vocational)	391 (30%)	283 (30%)	108 (29%)
High (high vocational/uni)	604 (47%)	453 (49%)	151 (41%)
Other	19 (1%)	12 (1%)	7 (2%)
Patient portal experience			
Yes / No	394 (30%) / 900 (70%)	272 (29%) / 657 (71%)	122 (33%) / 243 (67%)
Frequency of healthcare visits			
< 1 time per year	160 (12%)	132 (14%)	28 (8%)
1–4 times per year	517 (40%)	405 (44%)	112 (31%)
5–11 times per year	419 (32%)	281 (30%)	138 (38%)
1 time per month	75 (6%)	48 (5%)	27 (7%)
2–4 times per month	85 (7%)	49 (5%)	36 (10%)
> 1 time per week	38 (3%)	14 (2%)	24 (6%)
Health status			
Excellent	18 (1%)	17 (2%)	1 (0%)*
Very good	117 (9%)	106 (11%)	11 (3%)
Good	619 (48%)	517 (56%)	102 (28%)
Poor	441 (34%)	263 (28%)	178 (49%)
Bad	99 (8%)	26 (3%)	73 (20%)

### Generic portal preferences

Tables 3 and 4 show the overall results of the conjoint analysis, including all attributes and differences between the levels of the attributes. The most prominent and significant result is that respondents prefer to access a patient portal via a laptop or desktop above using a smartphone or tablet. Second, they prioritized to ask questions about medical data or about earlier visits to the provider via the portal. Thirdly, they dislike a delay of two weeks of their information shown in a portal as compared to direct publication of information, yet this is not a significant difference.

**Table 3:** General conjoint analysis of all respondents | Levels 2 and 3 compared to level 1

Attribute	Level	LogLike Diff.	*P*-Value
Accessibility	1. Portal can be accessed via a computer (laptop and/or desktop)	*	
	2. Portal can be accessed via a tablet (for example iPad)	−0.922	< 0.001
	3. Portal can be accessed via a smartphone (for example iPhone)	−1.086	< 0.001
Login	1. Username and password	*	
	2. DigiD with SMS verification	0.004	0.900
	3. Username, password and SMS verification	0.018	0.540
Interoperability	1. No export of data	*	
	2. Export of data to non-interoperable format (e.g. PDF)	0.259	< 0.001
	3. Export of data to interoperable format (e.g. Continuous Care Document)	0.165	< 0.001
Availability of information	1. Direct publication of info	*	
	2. Information delayed until discussed with provider	−0.024	0.460
	3. Available after 2 weeks independent if discussed with provider	−0.184	< 0.001
Content	1. Reports and basic information (e.g. medication overviews)	*	
	2. Reports and basic information and professional summary	0.312	< 0.001
	3. Complete uncensored medical file	0.303	< 0.001
Number of providers	1. Contains information about one provider (e.g. hospital or general practitioner)	*	
	2. Contains information about multiple providers (e.g. hospitals and general practitioner)	0.290	< 0.001
Patient-provider communication	1. No possibility to ask online questions	*	
	2. Possibility to ask questions about medical data in the portal or about earlier visits	0.684	< 0.001
	3. Online in-patient consult	0.539	< 0.001

* The asterisk indicates the base value

**Table 4:** General conjoint analysis of all respondents | Level 3 compared to level 2

Attribute	Level	LogLike Diff.	P-Value
Accessibility	2. Portal can be accessed via a tablet (for example iPad)	*	
	3. Portal can be accessed via a smartphone (for example iPhone)	20.32	< 0.001
Login	2. DigiD with SMS verification	*	
	3. Username, password and SMS verification	0.14	0.597
Interoperability	2. Export of data to non-interoperable format (e.g. PDF)	*	
	3. Export of data to interoperable format (e.g. Continuous Care Document)	5.48	0.001
Availability of information	2. Information delayed until discussed with provider	*	
	3. Available after 2 weeks independent if discussed with provider	9.82	< 0.001
Content	2. Reports and basic information and professional summary	*	
	3. Complete uncensored medical file	0.05	0.749
Patient-provider communication	2. Possibility to ask questions about medical data in the portal or about earlier visits	*	
	3. Online in-patient consult	13.93	< 0.001

* The asterisk indicates the base value

### Difference in preferences of subgroups

#### Medical condition and usage factors

Twenty-eight percent of the respondents were lung patients. Looking at the differences with the overall respondents and these lung patients, shown in Table 5, the overall respondents prefer a portal to contain information about multiple providers (e.g. hospitals and general practitioner), whereas the lung patients showed a small disfavor of this. A similar result appeared regarding which content to display in a portal. Overall respondents prefer reports, basic information and a professional summary, whereas lung patients prefer just reports and basic information, such as medication overviews.

**Table 5:** Conjoint analysis of sub groups | Levels 2 and 3 compared to level 1

Attribute	Level	LogLike Diff.				
		Total	Patient group	Age	Portal use experience	Gender
Accessibility	1. Portal can be accessed via a computer (laptop and/or desktop)	*				
	2. Portal can be accessed via a tablet (for example iPad)	−0.922	0.146	−0.301	0.376	−0.360
	3. Portal can be accessed via a smartphone (for example iPhone)	−1.086	0.228	−0.494	0.441	−0.549
Login	1. Username and password	*				
	2. DigiD with SMS verification	0.004	−0.064	−0.200	0.358	0.127
	3. Username, password and SMS verification	0.018	0.058	−0.124	0.236	0.038
Interoperability	1. No export of data	*				
	2. Export of data to non-interoperable format (e.g. PDF)	0.259	0.050	−0.217	0.297	−0.101
	3. Export of data to interoperable format (e.g. Continuous Care Document)	0.165	−0.008	0.169	0.335	0.140
Content	1. Reports and basic information (e.g. medication overviews)	*				
	2. Reports and basic information and professional summary	0.312	−0.031	−0.219	0.347	−0.099
	3. Complete uncensored medical file	0.303	0.069	−0.236	0.362	−0.045
Number of providers	1. Contains information about one provider (e.g. hospital or general practitioner)	*				
	2. Contains information about multiple providers (e.g. hospitals and general practitioner)	0.290	−0.018	−0.120	0.304	−0.113
Patient-provider communication	1. No possibility to ask online questions	*				
	2. Possibility to ask questions about medical data in the portal or about earlier visits	0.684	−0.003	−0.212	0.399	−0.158
	3. Online in-patient consult	0.539	0.005	−0.150	0.265	−0.039

* The asterisk indicates the base value

#### Age and usage factors

Of the overall respondents, 49% was aged 65+ and 46% was aged between 45 and 64 years old. Results of the subgroup analysis of patients aged < 65 are shown in Table 5. As the table shows, < 65 respondents prefer to access a portal via a laptop or desktop. They are less negative about using mobile devices compared to the overall respondent group, especially when it comes to using tablets. Patients aged < 65 further prefer secure login methods and do not necessarily want to have options for patient-provider communication via a portal.

#### Patient portal experience and usage factors

Thirty percent of the respondents had used a patient portal in their daily life and were thus experienced portal users. Table 5 shows that the experienced portal users are more positive towards using a smartphone or tablet to access a patient portal compared to the overall respondents. This accounts as well for the login means, experienced users are more positive towards using a more secure login means, such as a username, password and a verification code sent to a mobile phone.

#### Gender and usage factors

Of the overall respondents, 58% were male. In Table 5 it is reported that male respondents, similar to experienced portal users, are more positive regarding the use of mobile devices to access a patient portal compared to the overall respondents. Male respondents further prefer reports and basic information presented in a portal above a professional summary or a complete uncensored medical file. Male respondents likewise prefer to have information in a portal from one healthcare organization and do not necessarily want to have options for patient provider communication via a portal.

## Discussion

This study set out with the aim to value and prioritize patient portal usage factors reported by over 1200 cardiovascular patients and lung patients. It is interesting to note that the majority of our respondents (81%) were above 55 years old and 49% of our respondents were even aged 65 years and above. The results of this study show that our respondents prioritize user-friendly access to a portal, via a laptop or desktop, as well as being able to communicate with their provider via the portal to ask questions about their medical data or previous visits over other functionalities. Cardiovascular patients (72%) and lung patients (28%) differed in portal preferences regarding the medical information shown in a portal; lung patients prefer reports and basic information, such as medication overviews, and do not seem to require the option to contact a provider or to have a multiple provider overview.

### Access and login means in relation to older patients

The results of this study indicate that aging characteristics influence patient portal preferences, especially regarding technical aspects, such as access and login means. Our respondents state a preference to access a patient portal using a laptop or desktop, rather than using a tablet or a smartphone. Nevertheless, respondents aged younger than 65 years old, the majority being between 45 and 64 years old, were less negative about using a tablet as an access means than the overall respondent group. A possible explanation for older respondents’ access preference may be that the older adult and elderly target group had experience with inadequate user-interface designs of portals on small screens. Portals ─ both web-based and native app versions ─ have complex navigation structures. However, to suit the cognitive capacities of older patients and prevent usability problems, navigation complexities should be minimized [26, 27]. Furthermore, irrelevant information and cluttered presentation of (medical) information on smaller screens of tablets and smartphones inhibit older patients in reading and interpreting this information [28, 29]. A recent study has showed that older people increasingly do show interest in using tablets, yet they have concerns about the process of learning how to use such devices. They further worry about unclear instructions and support during that learning process [30]. Patient portal developers should take advantage of the older user interest in these devices. Albeit, in further development of mobile versions of patient portals, current knowledge on aging barriers influencing the experienced usability of mobile user-interface design [27] and portal functionalities should be taken into account.

Our analysis surprisingly showed that especially the elderly respondents preferred using a solely username and password as a login means to a patient portal. They preferred this above the more secure methods, called two factor authentication (2FA). Privacy and security are important aspects discussed in literature on patient preferences on patient portals [31–34] and the 2FA method is often mandated by governments to ensure privacy and security. The 2FA method is yet complex to use and often leads to usability problems experienced by older patients [35, 36]. We encourage software engineers in the field of privacy and security together with usability experts to rethink login means to patient portals in order to create a secure as well as a user-friendly login means. They can explore the opportunities for biometric authentication for example. In doing so, it is important to take the challenges of biometric authentication into account in relation to physical effects of older adults’ medical complications, such as cataracts and stroke [36]. An improved login means addressing both privacy and security as well as experienced ease of use by older patients, will likely strengthen their engagement in using patient portals.

### Publication of medical information

Respondents in our study were negative about a built-in publication delay of two weeks of their medical information. Nevertheless, our results show no significant difference in preferences between the options of ‘instant publication’ versus ‘publication after new information has been explained by a healthcare provider’. Previous studies evaluating the publication of medical information in patient portals show inconsistent results on whether publication empowers patients or if publication might harm patients when information is shown without mediating physician input [37, 38]. This is especially discussed within the perspective of publication of test results [37, 38]. Our study provides a strong indication that chronically ill patients do not prefer a delay in publication of their medical information in a patient portal. We therefore advise against such a delay feature in the implementation of portals of which chronically ill patients are the main user group. We further want to affirm the need for customization of medical information publication, where settings can be changed for each individual patient based on his/her preferences in obtaining medical information with or without mediating input from a physician.

The customizability of medical content in a portal is further emphasized in relation to the terminology used to publish this content in a portal. Most respondents prefer a summary of the medical information in laymen’s terms, presenting less but more understandable information, above a complete uncensored medical file. Our study therefore supports the idea that patients experience difficulties in understanding the medical information and jargon published in patient portals [31–33, 39–41]. In developing patient portals, we advise to consider customized features in which the provider can manually edit the content before publication. Another possible solution, better suited to the high workload of providers, is to automatically transform medicals terminology standards into laymen terms [42].

### Patient-provider communication

In our study, the option for using features to contact the healthcare provider is seen as a main priority by the respondents, which is in line with other studies claiming that such a functionality is an important facilitator for patient engagement [41, 43]. Patient-provider communication via a patient portal can yet be ineffective due to the absence or late replies from their physician [44]. This combination of findings suggest the need for further research on what patients define as a prompt response for various types of questions and how providers’ workflows can allow for such prompt responses on questions asked by patients via a portal.

### Patient portals and care across healthcare centers

An interesting finding of our study is that respondents showed a strong preference to use a portal with medical information from multiple providers, possibly working in various healthcare organizations. Especially experienced portal users preferred this, whereas male respondents were more in favor of obtaining medical information from one provider. Our results further show no preference for a digital interoperable export functionality and most respondents are interested in the option to create printable overviews such as in Word or PDF. Negative experiences with the cumbersome tasks of distributing health information across different centers, without any benefit of portals supporting this process, may be a possible explanation for this finding. This finding further shows that for chronically ill patients to gain more benefits from portals, the portal landscape needs to transform from ‘silos’ to an integrated ‘ecosystem’ of actors. In addition, it can be assumed that chronically ill patients currently manage their data across different healthcare centers by printing the data whereas they do want to have a holistic overview of their medical data across these centers in the future. Yet, they do not like to spend much effort in manually exporting digital information from one portal to another. In transforming portal silos to an integrated ecosystem, it is thus important to create systems that minimize the tasks of patients in creating a complete multicenter overview of their health data. A first step to achieve this is to technically facilitate the sharing of medical record information across centers by adopting interoperability standards, such as Fast Healthcare Interoperability Resources (FHIR) in the development of (future) digital environments for patients to access, manage and share their medical data [45].

### Limitations

This study has several limitations. First, respondents were part of a patient panel; we can therefore assume they have a high interest in aspects related to their health and disease management. Consequently, the findings of this study cannot be extrapolated to patients with a (very) low health status and/or interest in their health. These patients might have different preferences regarding patient portal design and use. In spite of this limitation, since our study is based on a large sample of respondents from two different chronically ill patient groups, this study certainly adds to our understanding of possible coherence of portal functionalities and usage factors in relation to disease management from the perspective of chronically ill patients. Furthermore, despite of the interest of the patient panels’ members in health and disease management, the majority of the respondents had no experience with patient portal use and were older people. Aspects that make them representatives of the Dutch chronically ill patient group. Second, it is possible that some words or formulations in the questionnaire were misunderstood by respondents or that respondents who had no prior experience in using a patient portal could not envision a portal based on the wording of the questionnaire. This problem was limited by validating the questionnaire before using it in practice. Third, the underlying model for a choice-based conjoint experiment is nonlinear due to the modeling using a logit function. The variance-covariance matrix which is used to generate the design is dependent on betas. Since we had no prior knowledge about these betas we chose to assume linearity just as rating-based methods and developed the choice sets using heuristics. In order to gain a more optimal design a pre-test or assuming a prior distribution would have improved the outcomes and statistical efficiency.

## Conclusions

The current study found that preferred technical aspects by our older patient respondents, such as patient portal access via a laptop, secure login means and being able to export data via Word of PDF, are similar and independent of a specific medical condition. Yet, lung patients and heart and vascular patients do vary when it comes to preferences on usage factors related to publication of medical content and digital patient-provider communication means via a portal. It is therefore highly assumable that offering solid and user-friendly access as well as a consistent technical basis of functionalities across the variety of patient portals, could help increase patient portal acceptance by older patients; ultimately helping to stimulate patient engagement via patient portal use. By researching underlying reasons to preferences on patient portal functionalities and usage factors, future studies can gain more understanding of how to adjust patient portals to the needs of patients with specific or multiple medical conditions and their distinguishing patient journeys.

## Additional files

Additional file 1: Search strategy. The search strategy for the literature review performed to define the attributes and levels to be used in the conjoint analysis. (DOCX 19 kb)

Additional file 2: Overview of included studies. List of included studies of the literature review performed to define the attributes and levels to be used in the conjoint analysis. (DOCX 81 kb)

Additional file 3: Questionnaire. The questionnaire used for the conjoint analysis choice experiment. Provided are 1) the original version of the questionnaire in Dutch and 2) a translated version of the questionnaire in English for the readers of this article. (DOCX 2549 kb)

## Abbreviations

2FA: Two factor authentication
EHR: Electronic Health Record
FHIR: Fast Healthcare Interoperability Resources
HVG: Heart and Vascular Group (Heart Council)
LF: Lung Fund
