# Supplementary material for: How do patients value and prioritize patient portal functionalities and usage factors? A conjoint analysis study with chronically ill patients
Source: BMC Med Inform Decis Mak. 2018 Nov 21;18:108. doi: 10.1186/s12911-018-0708-5 (PMC6249922; doi:10.1186/s12911-018-0708-5)
Supplement: Supplementary file 1 — Search strategy. The search strategy for the literature review performed to define the attributes and levels to be used in the conjoint analysis. (DOCX 19 kb) [file 12911_2018_708_MOESM1_ESM.docx]

**Appendix A - Search strategy**

To perform the literature review, two independent searches were conducted in two databases: Ovid and the Cochrane library. A predefined search strategy was used in both databases between November 15th and the 6th of December 2016. The search in the Cochrane library served as an orienting search to find high level evidence such as systematic reviews.
To build the search query used in the second search, keywords were extracted from the references and supplemented by performing an explorative search on Google (Scholar), PubMed and elicited from key papers. The second search was performed in Ovid and search results from this search were used to in- and exclude studies. Table 1 provides the used search queries for the first and second search.

Table 1 - Search queries of first and second search

| First search  **Database:** Cochrane  **Goal:** orienting, form correct search terms for second search | ((online portal) **or** (patient portal) **or** (personal health record)) |
| --- | --- |
| Second search  **Database:** Ovid  **Goal:** study in/exclusion and data analysis with included studies | (((online portal **or** personal health records **or** ((portal **or** portals) adj5 (patient **or** patients)) **or** (health adj3 record adj3 (patient **or** patients))) and ((engagement adj5 (patient **or** patients)) **or** implementation **or** facilitator* **or** barrier* **or** (acceptance adj5 (patient **or** patients)) **or** adoption **or** (perception* adj5 (patient **or** patients)) **or** (satisfaction adj5 (patient **or** patients)) **or** ((experience **or** experiences) adj5 (patient **or** patients)) **or** (perspective* adj5 (patient **or** patients)) **or** willingness **or** need **or** needs **or** demand **or** demands **or** tethered **or** implication **or** implications **or** (electronic adj5 (patient **or** patients)))) not (portal vein **or** hepatic vein **or** portal hypertension)) |

Inclusion criteria were: 1) availability of English full text version; 2) article studied a patient portal; 3) study objectives were to describe possible (subjective) factors which could influence patients’ usage. Exclusion criteria were: 1) study reported quantitative outcomes (e.g. number of telephone calls, clinical outcome, and number of online messages) 2) study reported solely on overall satisfaction of patients’ portal usage. Articles were first screened on title and abstract. Subsequently, articles were screened on full text. Results from included studies were discussed with two other researchers to check for validity and completeness. Screenings were performed by two independent reviewers to increase screening reliability. Consensus was reached by discussing the results. Quality assessment of articles was performed by constructing a predefined appraisal list, including publication year, country, study design, key participant characteristics, setting, measurements, portal type, main functionalities of portal defined by article, possible influencing factors on use, and severe limitations.
