# Supplementary material for: How do patients value and prioritize patient portal functionalities and usage factors? A conjoint analysis study with chronically ill patients
Source: BMC Med Inform Decis Mak. 2018 Nov 21;18:108. doi: 10.1186/s12911-018-0708-5 (PMC6249922; doi:10.1186/s12911-018-0708-5)
Supplement: Supplementary file 2 — Overview of included studies. List of included studies of the literature review performed to define the attributes and levels to be used in the conjoint analysis. (DOCX 81 kb) [file 12911_2018_708_MOESM2_ESM.docx]

## Appendix B - Overview of included studies

The Ovid search resulted in 1.229 resulted in unique articles. After title and abstract screening 115 articles remained for full-text screening. After this second screening, 38 articles met all inclusion criteria and were included for analysis. Most common reasons for exclusion during full text screening were; articles did not discuss any barriers/facilitators (n=10), only objectives measures were presented (n=8) or the portal was managed by a third party (e.g. patient or insurer) (n=18). The flow diagram displayed in Figure 1 shows a graphic overview of the selection process. Table 1 and 2 provide the study characteristics and reported portal functionalities and influencing factors of the included studies.


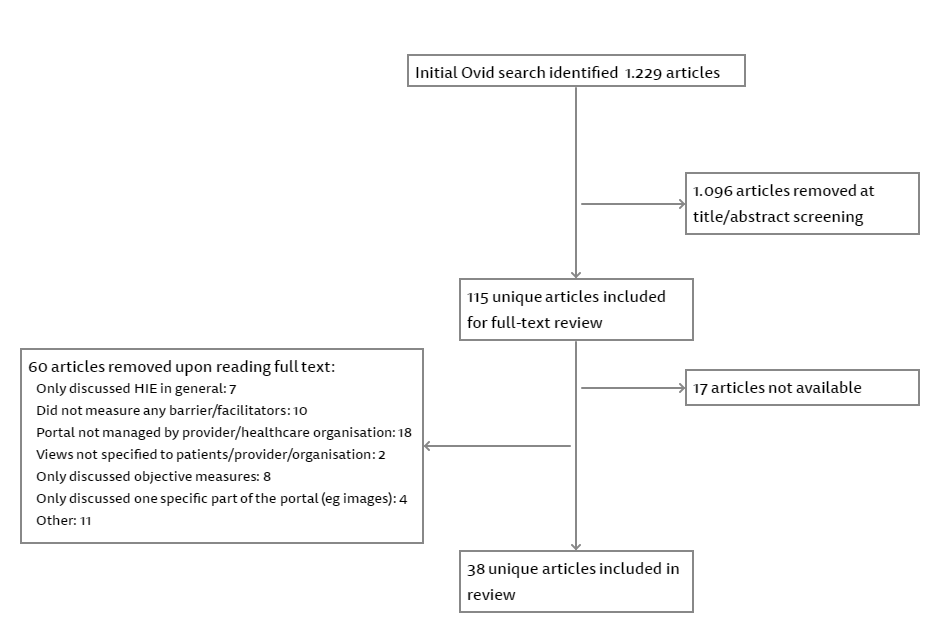


Figure 1: Flow diagram study selection

Table 1 - Study characteristics of included studies

| **no.** | **Author** | **Year** | **Country** | **Study design** | **Sample size** | **Portal** |
| --- | --- | --- | --- | --- | --- | --- |
| 1 | White | 2016 | United Kingdom | Cross-sectional study using self-completed questionnaire | 209 | No portal used |
| 2 | Kamo | 2016 | United states | Description of system | 0 | MyVirginiaMason |
| 3 | Lyles | 2016 | United states | Qualitative study using ten focus groups | 87 | kp.org |
| 4 | Gagnon | 2015 | Canada | Qualitative using semi-structured interviews | 35 | No portal used |
| 5 | Sorondo | 2016 | United states | Qualitative prospective focus groups and structured interview | 92 | Kryptiq CareCatalyst |
| 6 | Vydra | 2015 | United states | Qualitative study using focus groups | 5 | Mychart |
| 7 | Tieu | 2015 | United states | Qualitative study using focus groups | 16 | No portal used |
| 8 | Reicher | 2015 | United states | Description of system | 16.101 | kp.org |
| 9 | Ammerlaan | 2015 | Netherlands | Qualitative study using semi-structured interviews | 13 | Reumaportaal |
| 10 | Black | 2015 | United states | Qualitative study using focus groups | 31 | Mychart |
| 11 | Ålander | 2015 | Sweden | Qualitative study using questionnaire | 3266 | My Healthcare Contacts |
| 12 | Ronda | 2015 | Netherlands | Qualitative study using questionnaire | 632 | Digitaal Logboek |
| 13 | Bush | 2015 | United states | Qualitative study using structured telephone interviews | 9 | Mychart |
| 14 | Clark | 2015 | United states | Qualitative study using questionnaire | 1420 | No portal used |
| 15 | Otte-trojel | 2015 | Netherlands | Qualitative study using interviews | 10 | Multiple |
| 16 | Hassol | 2004 | United states | Qualitative study using questionnaires and two focus groups | 1.421 (questionnaire) 25 (focus group) | Mychart |
| 17 | Mcnamara | 2014 | United states | Qualitative study using questionnaire | 41 | No portal used |
| 18 | Latulipe | 2015 | United states | Qualitative study using semi-structured interviews | 52 | No portal used |
| 19 | Tieu | 2016 | United states | Qualitative study using semi-structured interviews | 25 | MYSFHEALTH |
| 20 | Luque | 2013 | United states | Qualitative study using questionnaires and focus groups | 90 (questionnaire)  8 (focus group) | No portal used |
| 21 | Alpert | 2016 | United states | Qualitative study using questionnaires and two focus groups | 31 (questionnaire) 13 (focus group) | MyPreventiveCare |
| 22 | Turner | 2015 | United states | Qualitative study using semi-structured interviews | 74 | MyChart |
| 23 | Grunloh | 2016 | Sweden | Qualitative study using structured interviews | 12 | My Healthcare Contacts |
| 24 | Nguyen | 2016 | Canada | Qualitative study using four focus groups | 29 | No portal used |
| 25 | Harrison | 2015 | Canada | Qualitative study using questionnaire | 63 | No portal used |
| 26 | Mishuris | 2014 | United states | Qualitative study using semi-structured interviews | 16 | My HealtheVet |
| 27 | Milller | 2016 | United states | Qualitative study using semi-structured interviews | 20 | Multiple |
| 28 | Hess | 2008 | United states | Qualitative study using two focus groups (pre-post implementation) | 39 | UPMC HealthTrak |
| 29 | Mayberry | 2011 | United states | Qualitative study using focus groups | 75 | My-HealthAtVanderbilt |
| 30 | Wells | 2014 | New Zealand | Qualitative study using structured telephone interviews | 30 | Multiple |
| 31 | Ronda | 2015 | Netherlands | Qualitative study using questionnaire | 12.793 | Digitaal Logboek |
| 32 | Zarcadoolas | 2013 | United states | Qualitative study using focus groups | 28 | No portal used |
| 33 | Goel | 2011 | United states | Qualitative study using structured telephone interviews | 159 | My chart |
| 34 | Gee | 2015 | United states | Qualitative study using semi-structured interviews | 18 | Multiple |
| 35 | Britto | 2013 | United states | Qualitative study using semi-structured interviews | 24 | Multiple |
| 36 | Woods | 2013 | United states | Qualitative study using focus group interviews. | 36 | My HealtheVet |
| 37 | Yau | 2011 | Canada | Qualitative study using semi-structured interviews | 10 | mydoctor.ca |
| 38 | Dhanireddy | 2012 | United states | Qualitative study using focus groups | 30 | No portal used |

Table 2 – Reported portal functionalities and influencing factors per included study

| **no.** | **Author** | **Main functionalities defined by article** | **Influencing factors** |
| --- | --- | --- | --- |
| 1 | White | Not applicable | Need for engagement; Health literacy/ numeracy skills; Alignment of workflow /increase of workload |
| 2 | Kamo | Appointment requests and self-schedule; Clinical messaging with providers; Test results; Medications; Allergies; Upcoming appointments; Medication refill requests | Health literacy/ numeracy skills; Proxy access; Accessibility/completeness; Limited access to the Internet / computer; Timeliness; Alignment of workflow /increase of workload |
| 3 | Lyles | Viewing medical history; Visit summaries; Immunizations; Allergies, Laboratory results; Prescription renewals; Appointment requests; Clinical messaging with providers | Health literacy/ numeracy skills; Computer/technical skills / IT literacy; Interfere with personal relationships; Prefer talking to real person; Conservative; Security/ privacy; Registration & login process; Difficult medical content; Need for training/support |
| 4 | Gagnon | Not applicable | Health literacy/ numeracy skills; Accessibility/completeness; Limited access to the Internet / computer; Computer/technical skills / IT literacy; Conservative; Security/ privacy; (Perceived) usefulness; Support from professional; Costs/ Usage fees; Cross-platform software; Difficult medical content; Need for training/support; User interface customization; Interoperability; Remuneration; Restricted patient control of data |
| 5 | Sorondo | Problem list, Medications; Laboratory and radiology results; Appointment requests; Prescription renewals; Obtaining referrals. | Prefer talking to real person; Security/ privacy; (Perceived) usefulness; Support from professional; Technical problems; Need for training/support; Remuneration |
| 6 | Vydra | Not reported in article | Alignment of workflow /increase of workload; Training and education; Remuneration; Improved patient satisfaction |
| 7 | Tieu | Not applicable | easier means of communication; Possibility to improve the effectiveness of in-patient consult; Limited access to the Internet / computer; Proxy access; Computer/technical skills / IT literacy; Communication supplement; Interfere with personal relationships; Security/ privacy; Registration & login process; Difficult medical content |
| 8 | Reicher | Basic healthcare information; Optional access to radiology results; Clinical messaging with providers | Accessibility/completeness; Technical problems; Rapid access to new data |
| 9 | Ammer-laan | E-consult; Clinical notes; Laboratory results; Upcoming appointments; Online self-monitoring | Involvement of others; Easier means of communication; Possibility to improve the effectiveness of in-patient consult; Need for engagement; Cognitive overload; Security/ privacy; (Perceived) usefulness; Registration & login process |
| 10 | Black | Upcoming appointments; Lab results; Requesting or canceling appointments; Prescription renewals; Past AVS forms | Limited access to the Internet / computer; Computer/technical skills / IT literacy; Interfere with personal relationships; Security/ privacy; (Perceived) usefulness; Support from professional; Lack of awareness; Expectations vs experience; Alignment of workflow /increase of workload; |
| 11 | Ålander | Appointment requests and self-schedule; Request certificates; Basic medical data information; Extend sick leave; Clinical messaging with providers; Update personal data; Change house physician/family doctor; Prescription renewals and assistive tools; Order a written copy of the medical health record | Proxy access; Difficult medical content |
| 12 | Ronda | Clinic notes; Results physical examination; Laboratory results; Problem lists and treatment goals; Medications; General diabetes information | Communication supplement; Lack of awareness |
| 13 | Bush | Clinical messaging with providers; Appointment scheduling; Result reporting; Health information | Security/ privacy; (Perceived) usefulness; Registration & login process; Need for training/support |
| 14 | Clark | Not applicable | Need for engagement; Timeliness; Security/ privacy; Lack of awareness |
| 15 | Otte-trojel | Not reported in article | Health literacy/ numeracy skills; Alignment of workflow /increase of workload; Interoperability; Cost |
| 16 | Hassol | View of 25 frequently ordered laboratory tests and explanation of the results; Allergies; medications; Problem list; Past/Upcoming appointments; Health-related histories; Clinical messaging with providers; Appointment requests; Prescription renewals; Request referrals | Easier means of communication; Accessibility/completeness; Proxy access; Security/ privacy; Registration & login process; Difficult medical content; Restrict patient to access the data/ patient anxiety |
| 17 | Mcnamara | Not applicable | Possibility to improve the effectiveness of in-patient consult; Restrict patient to access the data/ patient anxiety |
| 18 | Latulipe | Not applicable | Accessibility/completeness; Limited access to the Internet / computer; Computer/technical skills / IT literacy; Communication supplement; Interfere with personal relationships; Prefer talking to real person; Conservative; Security/ privacy; (Perceived) usefulness; Registration & login process; Difficult medical content; Need for training/support |
| 19 | Tieu | Not reported in article | Computer/technical skills / IT literacy; Difficult medical content |
| 20 | Luque | Not applicable | Health literacy/ numeracy skills; Limited access to the Internet / computer; Computer/technical skills / IT literacy; Conservative; Security/ privacy; Costs/ Usage fees |
| 21 | Alpert | Laboratory results; Viewing information from the medical record, personalized recommendations | Accessibility/completeness; Communication supplement; Interfere with personal relationships; (Perceived) usefulness; Registration & login process; Difficult medical content |
| 22 | Turner | Not reported in article | Need for engagement; Limited access to the Internet / computer; Conservative; Security/ privacy; Registration & login process |
| 23 | Grunloh | Health care information; Test results; 10 eHealth services such as appointment scheduling, following referrals, Information about which provider accessed the medical record | Possibility to improve the effectiveness of in-patient consult; Communication supplement; Difficult medical content; Alignment of workflow /increase of workload; Improved patient satisfaction; Restrict patient to access the data/ patient anxiety; Fear of control |
| 24 | Nguyen | Not reported in article | Easier means of communication; Need for engagement; Health literacy/ numeracy skills; Computer/technical skills / IT literacy; Communication supplement; Security/ privacy; (Perceived) usefulness; Costs/ Usage fees; Difficult medical content; User interface customization |
| 25 | Harrison | Not reported in article | Communication supplement; Limited access to the Internet / computer; Security/ privacy; (Perceived) usefulness; Restrict patient to access the data/ patient anxiety |
| 26 | Mishuris | Download medical record; Clinical messaging with providers; Prescription renewals; Input data about health; Generic health information; Education tools | Easier means of communication; Limited access to the Internet / computer; (Perceived) usefulness; Support from professional; Lack of awareness; Need for training/support; Training and education |
| 27 | Milller | Not reported in article | Easier means of communication; Health literacy/ numeracy skills; Proxy access; Computer/technical skills / IT literacy; Communication supplement; Security/ privacy; Alignment of workflow /increase of workload; Improved patient satisfaction; Restrict patient to access the data/ patient anxiety; Easier means for communication; Low uptake |
| 28 | Hess | Test results; Medications; Problem lists; Health reminders; Communicate electronically; Education tools; User entered health data | Easier means of communication; Need for engagement; Accessibility/completeness; Communication supplement; Rapid access to new data; Registration & login process; |
| 29 | Mayberry | Not reported in article | Support from professional; Proxy access; Support from kin; Lack of awareness; Cross-platform software; Need for training/support |
| 30 | Wells | Not reported in article | Communication supplement; Lack of awareness; Alignment of workflow /increase of workload; Training and education; Remuneration; organizational vision |
| 31 | Ronda | Clinic notes; Results physical examination; Laboratory results; Problem lists and treatment goals; Medications; General diabetes information | Conservative; Lack of awareness |
| 32 | Zarcadool-as | Not applicable | Easier means of communication; Health literacy/ numeracy skills; Proxy access; Communication supplement; Prefer talking to real person; Security/ privacy; (Perceived) usefulness |
| 33 | Goel | Upcoming appointments; Laboratory results; Appointment scheduling; Prescription renewals; Viewing past AVS forms | Limited access to the Internet / computer; Communication supplement; Conservative; Security/ privacy; Lack of awareness; Registration & login process |
| 34 | Gee | Not reported in article | Possibility to improve the effectiveness of in-patient consult; Need for engagement; Health literacy/ numeracy skills; Timeliness; Communication supplement; Security/ privacy; Difficult medical content; Need for training/support; User interface customization; Accessibility/completeness |
| 35 | Britto | Demographic and contact information; Test results; Medications; Clinical messaging with providers | Interfere with personal relationships; Prefer talking to real person; Easier means of communication; Communication supplement; Difficult medical content |
| 36 | Woods | Clinic notes; Hospital discharge notes; Problem lists; Vital signs; Medications; Allergies; Appointments; Laboratory and imaging results; Education tools; Proxy access, User entered health data | Involvement of others; Possibility to improve the effectiveness of in-patient consult; Accessibility/completeness; Communication supplement; Inconsistencies in Content; Difficult medical content |
| 37 | Yau | Not reported in article | Security/ privacy; Difficult medical content; Alignment of workflow /increase of workload; Remuneration; Restrict patient to access the data/ patient anxiety; Fear of control |
| 38 | Dhanire-ddy | Not applicable | Easier means of communication; Possibility to improve the effectiveness of in-patient consult; Need for engagement; Cognitive overload; Accessibility/completeness; Communication supplement; Interfere with personal relationships; Prefer talking to real person; Security/ privacy; (Perceived) usefulness; Support from professional; Difficult medical content |
