# Supplementary material for: How do patients value and prioritize patient portal functionalities and usage factors? A conjoint analysis study with chronically ill patients
Source: BMC Med Inform Decis Mak. 2018 Nov 21;18:108. doi: 10.1186/s12911-018-0708-5 (PMC6249922; doi:10.1186/s12911-018-0708-5)
Supplement: Supplementary file 3 — Questionnaire. The questionnaire used for the conjoint analysis choice experiment. Provided are 1) the original version of the questionnaire in Dutch and 2) a translated version of the questionnaire in English for the readers of this article. (DOCX 2549 kb) [file 12911_2018_708_MOESM3_ESM.docx]

**Appendix C - Questionnaire**

In appendix C we first show screenshots of an example of the original digital questionnaire in Dutch. Then we provide the text of the questionnaire in English.

Please note: 1) only the Dutch questionnaire has been send to the patient panels. The English text is made for this article to provide sight on the questionnaire items for non-Dutch speakers. Due to the translation into English the meaning of the text might be interpreted differently. For questions about the meaning of the original Dutch version of the questionnaire please contact the corresponding author. 2) The question on which portal the respondent prefers is asked for each questions. To show the phrasing of the question, we placed it at question 1, yet not at the other boxes.


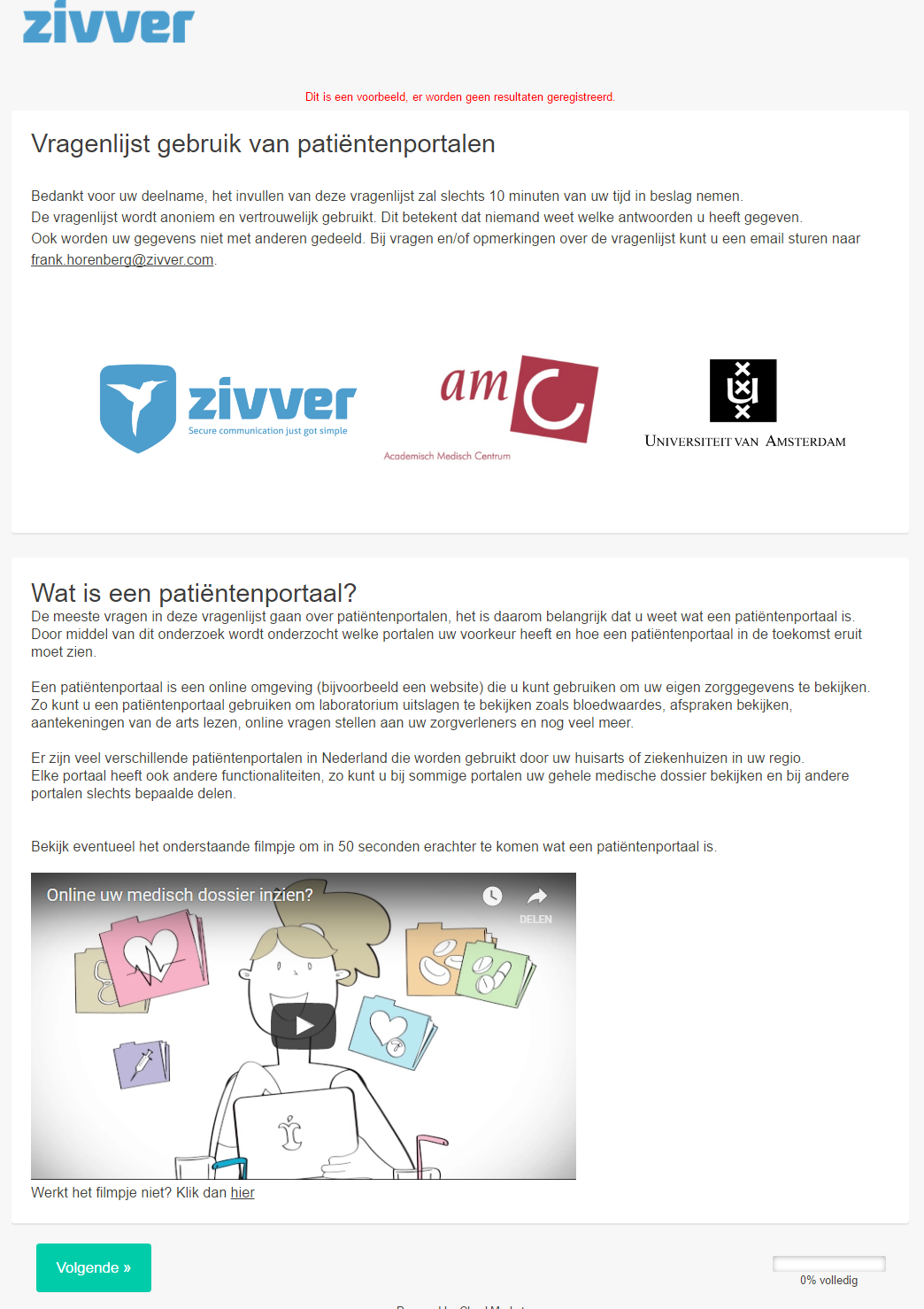


Research Institute Logo 1

Research Institute Logo 1

Research Institute

Logo 2

Research Institute

Logo 3


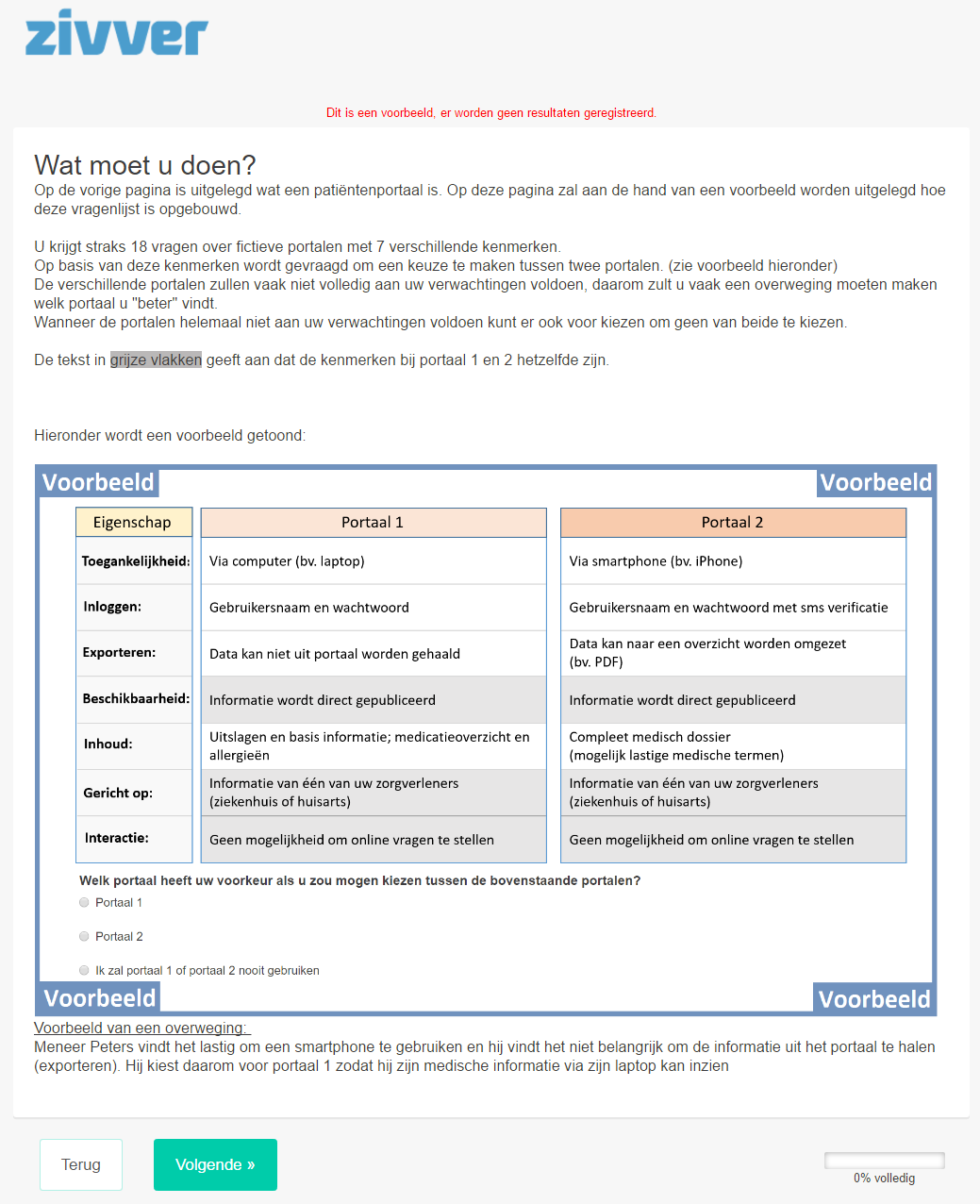


Research Institute Logo 1


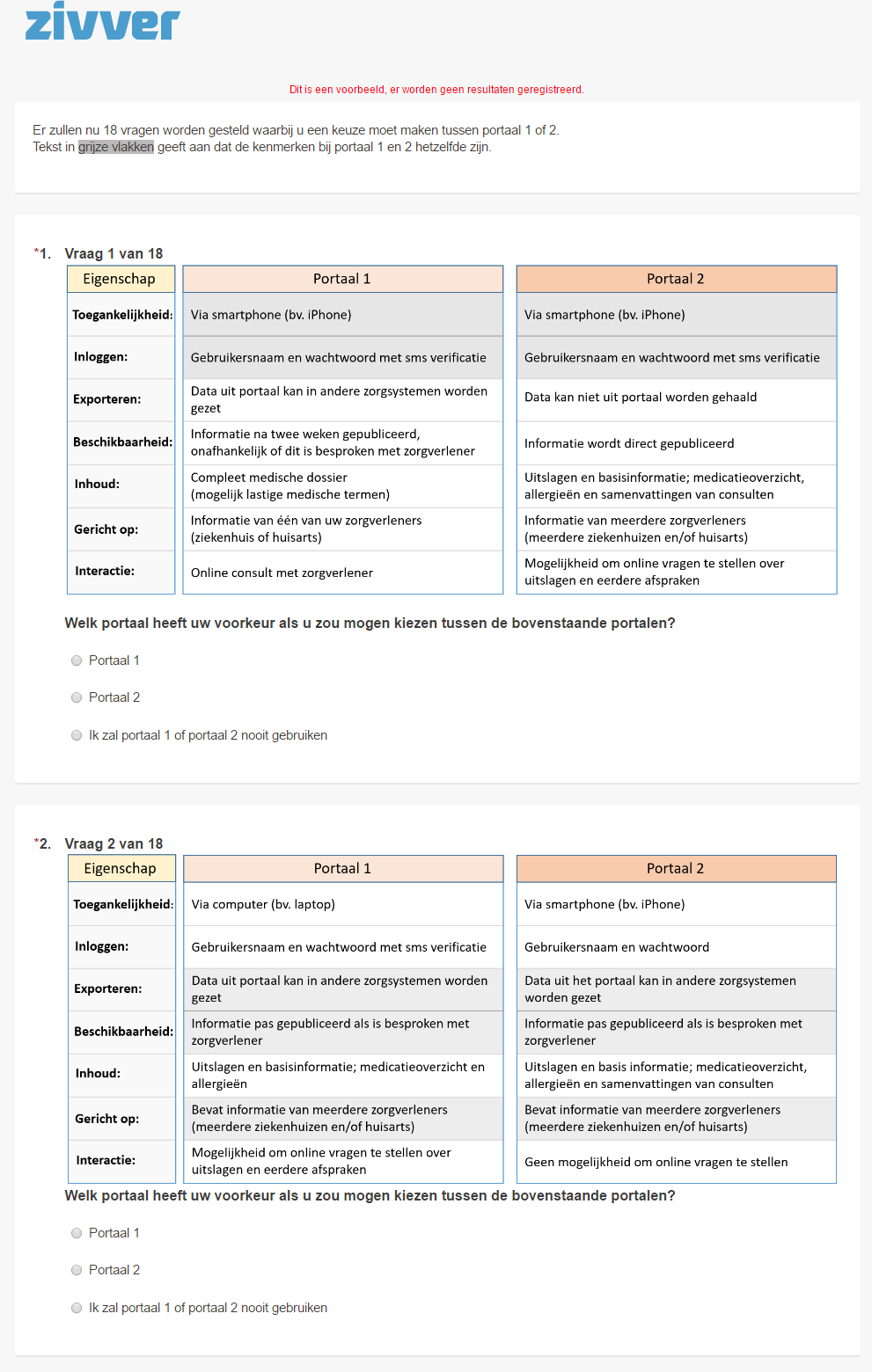


Research Institute Logo 1


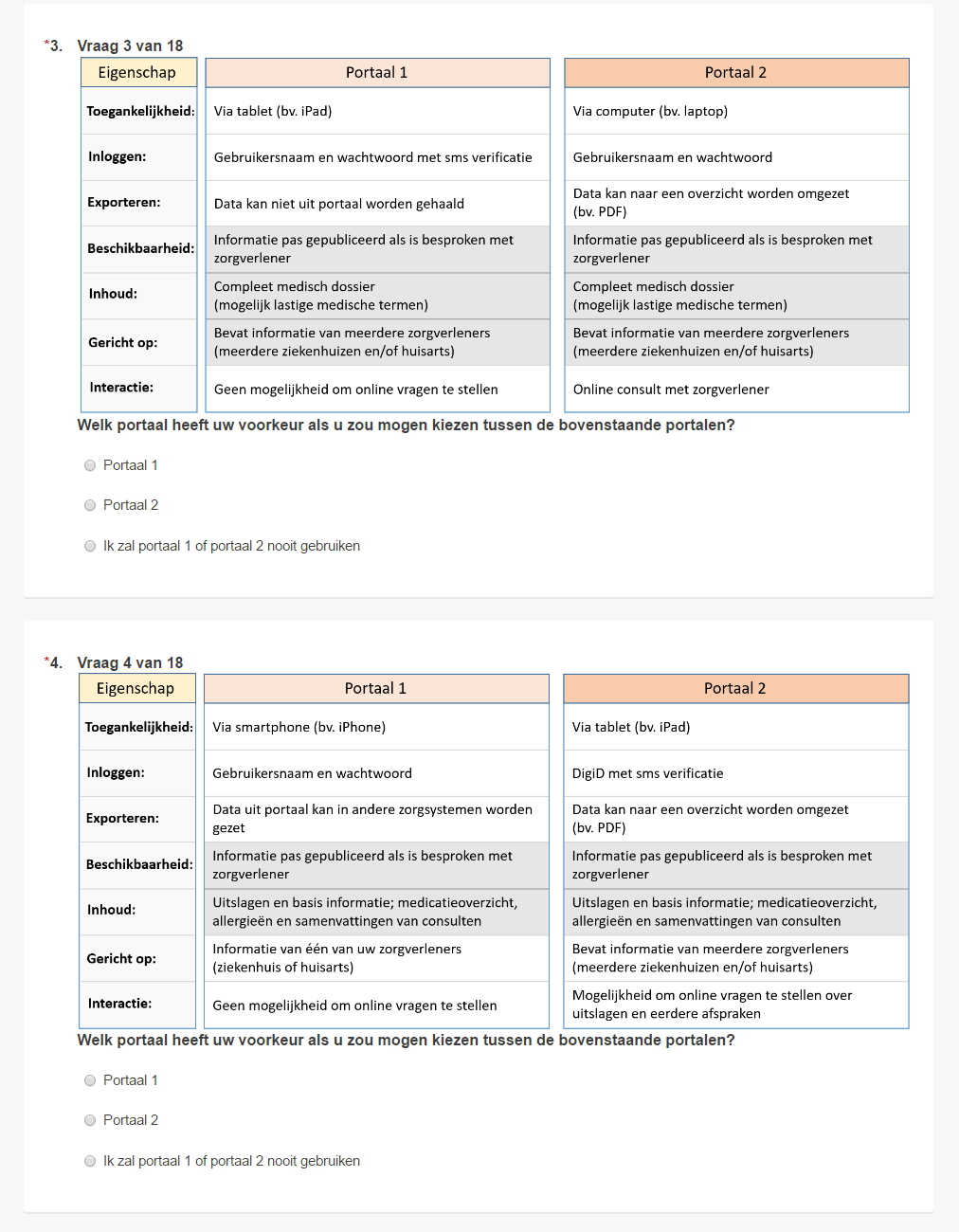


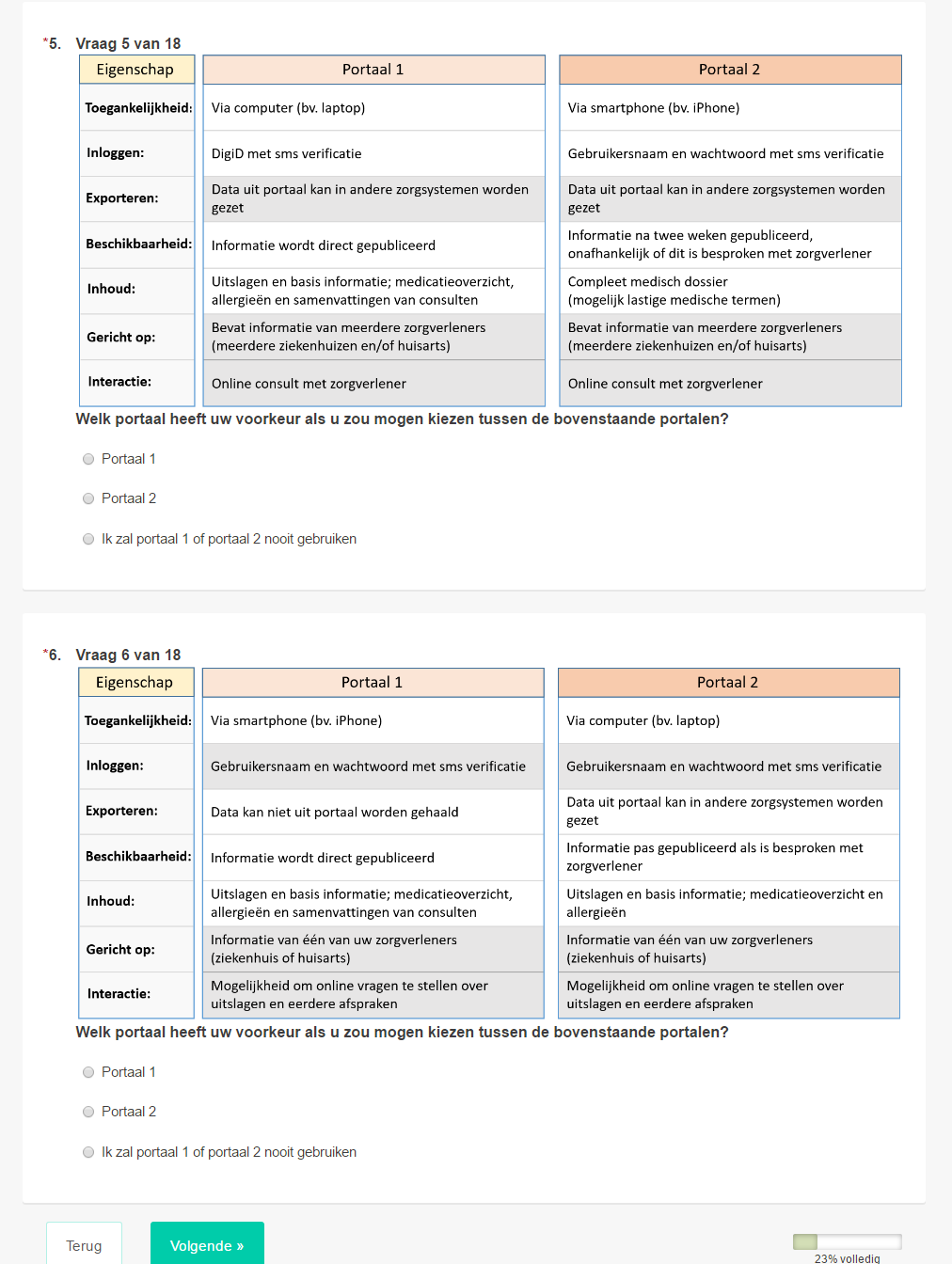

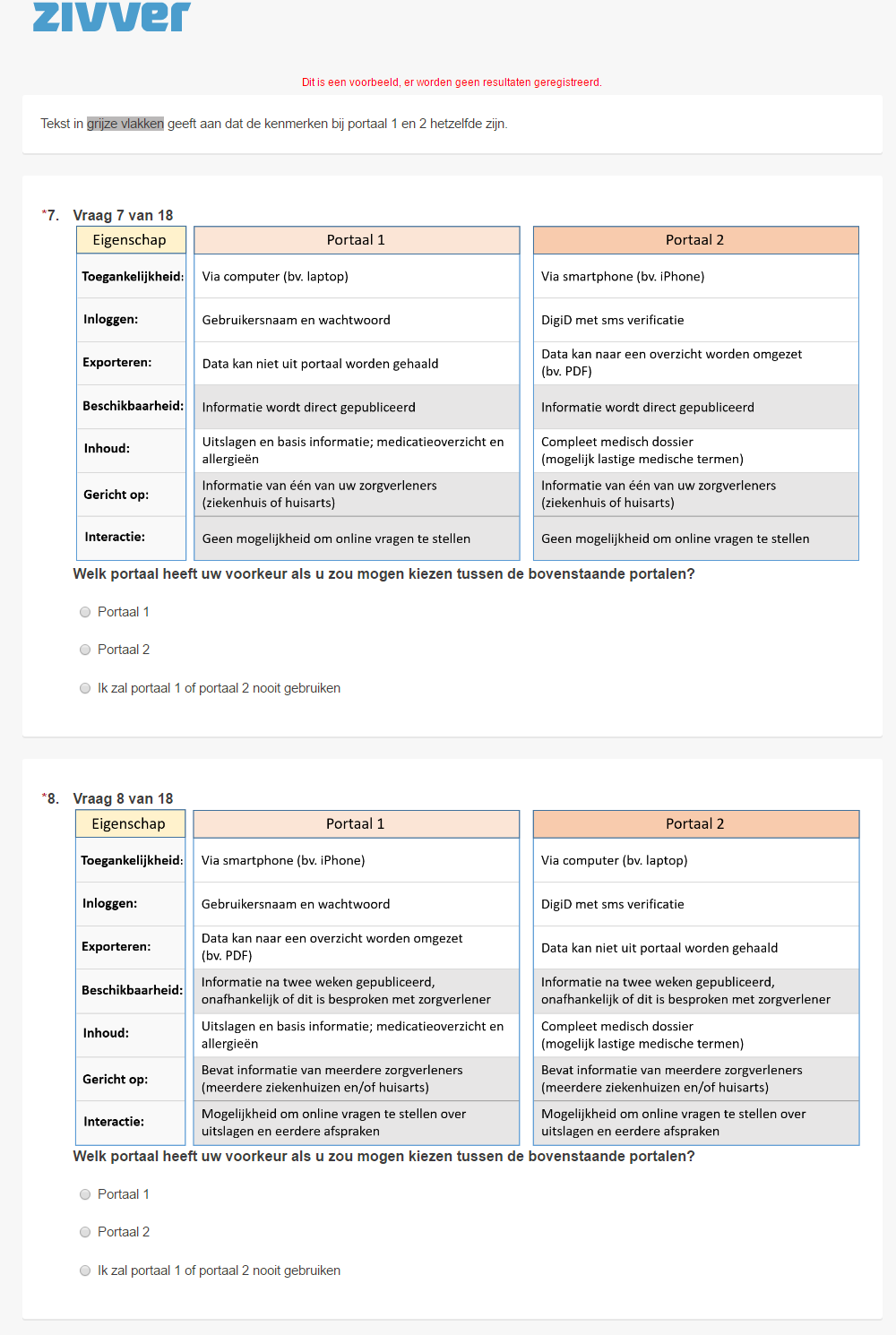

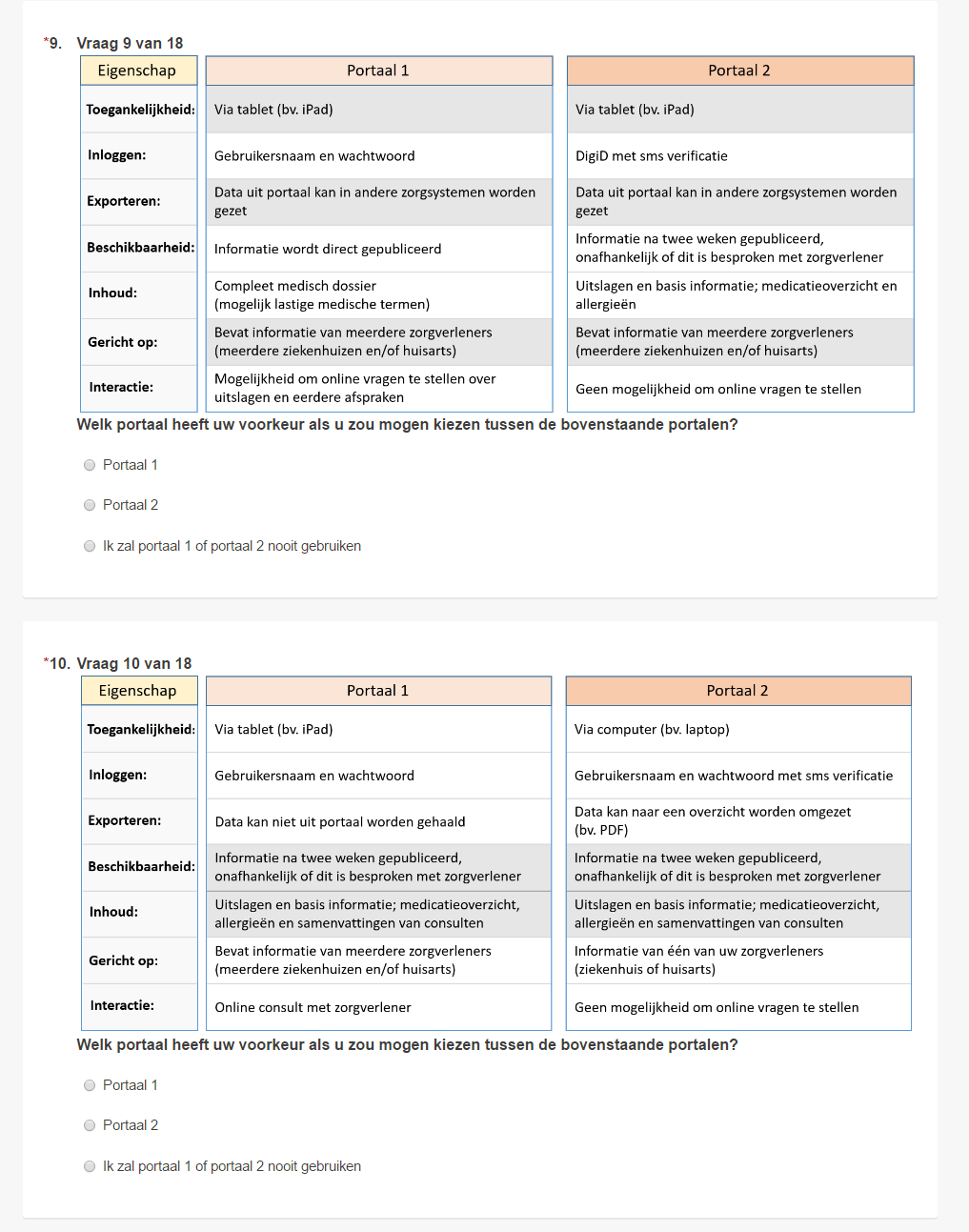

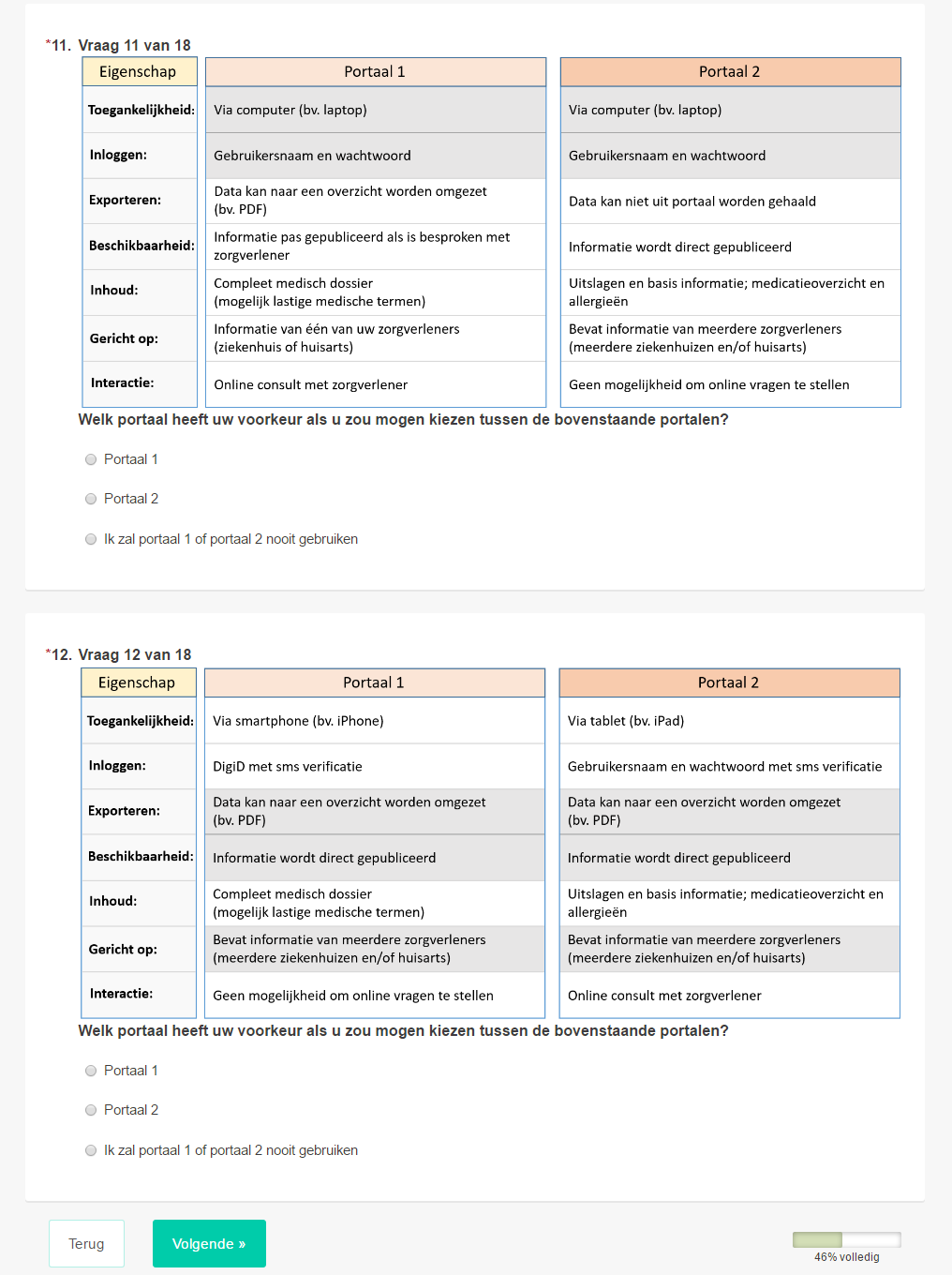

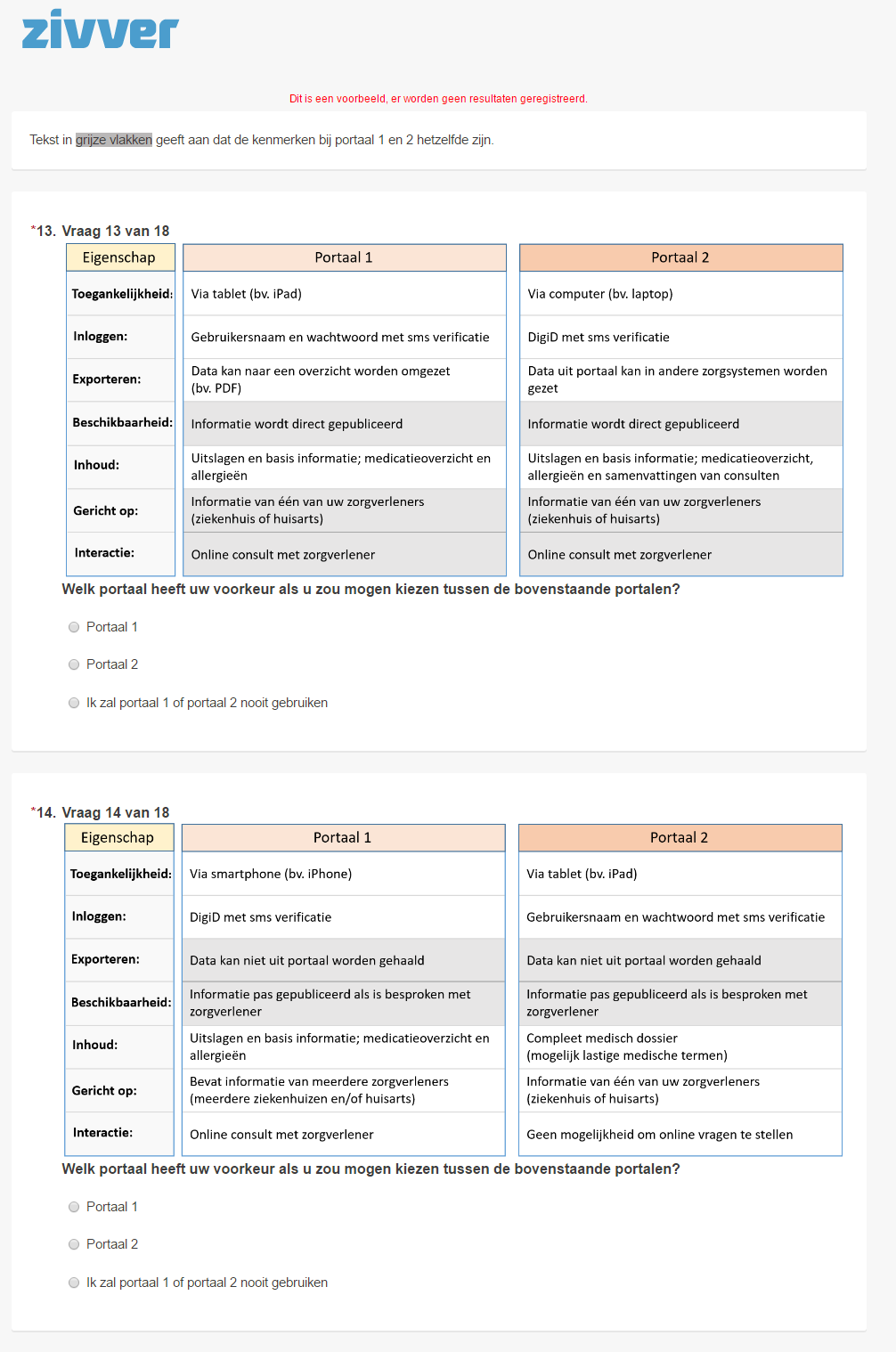

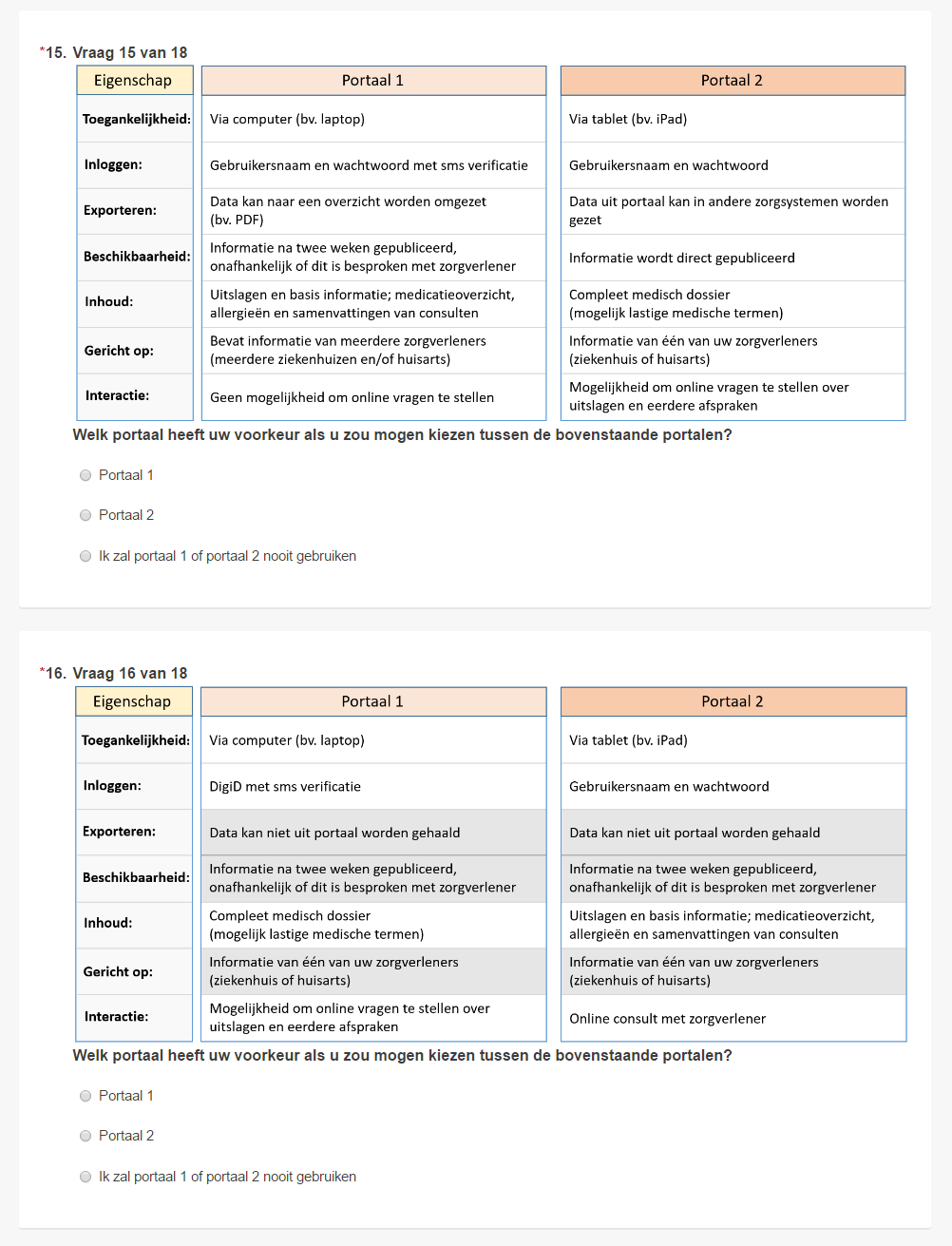

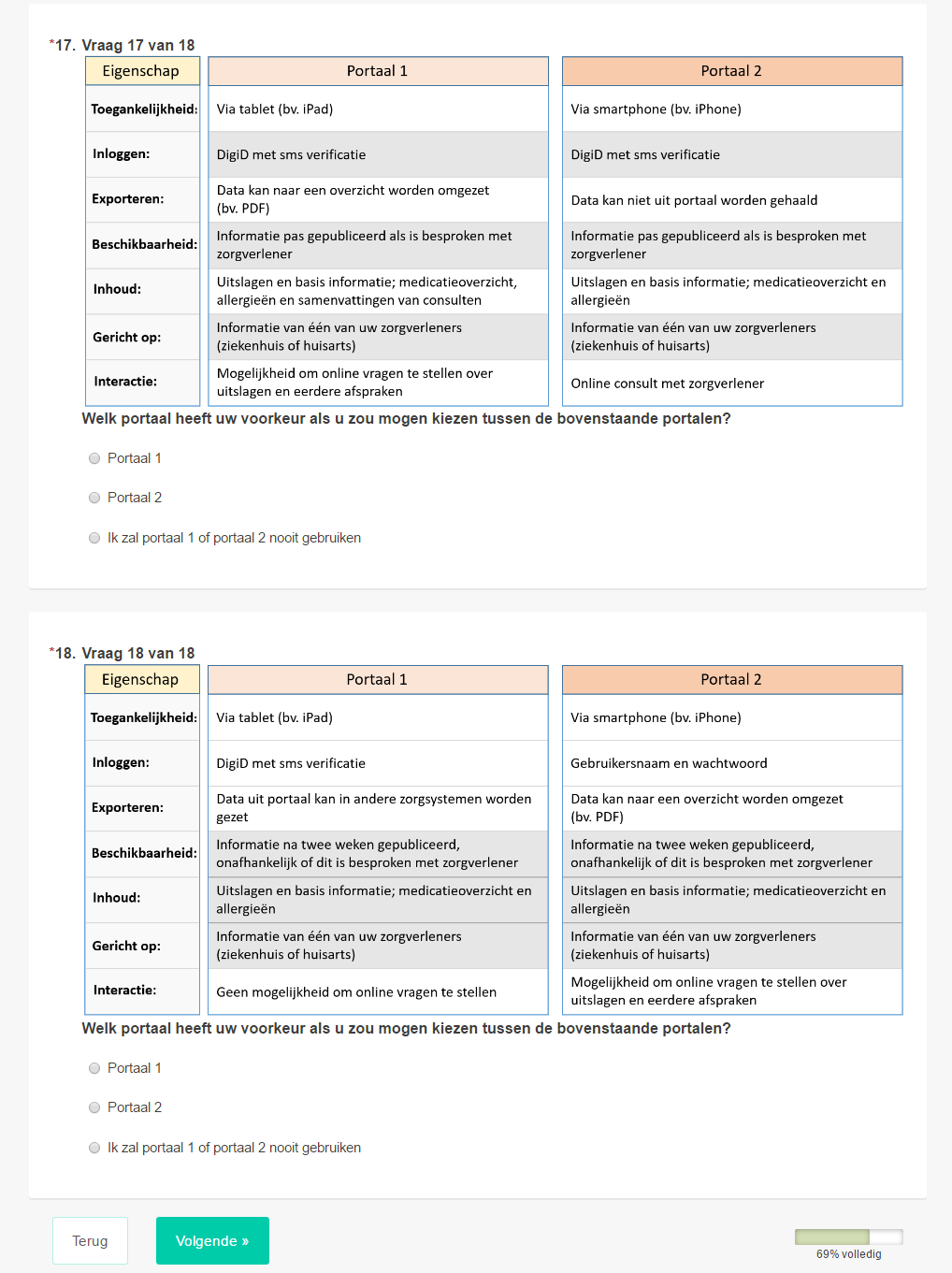

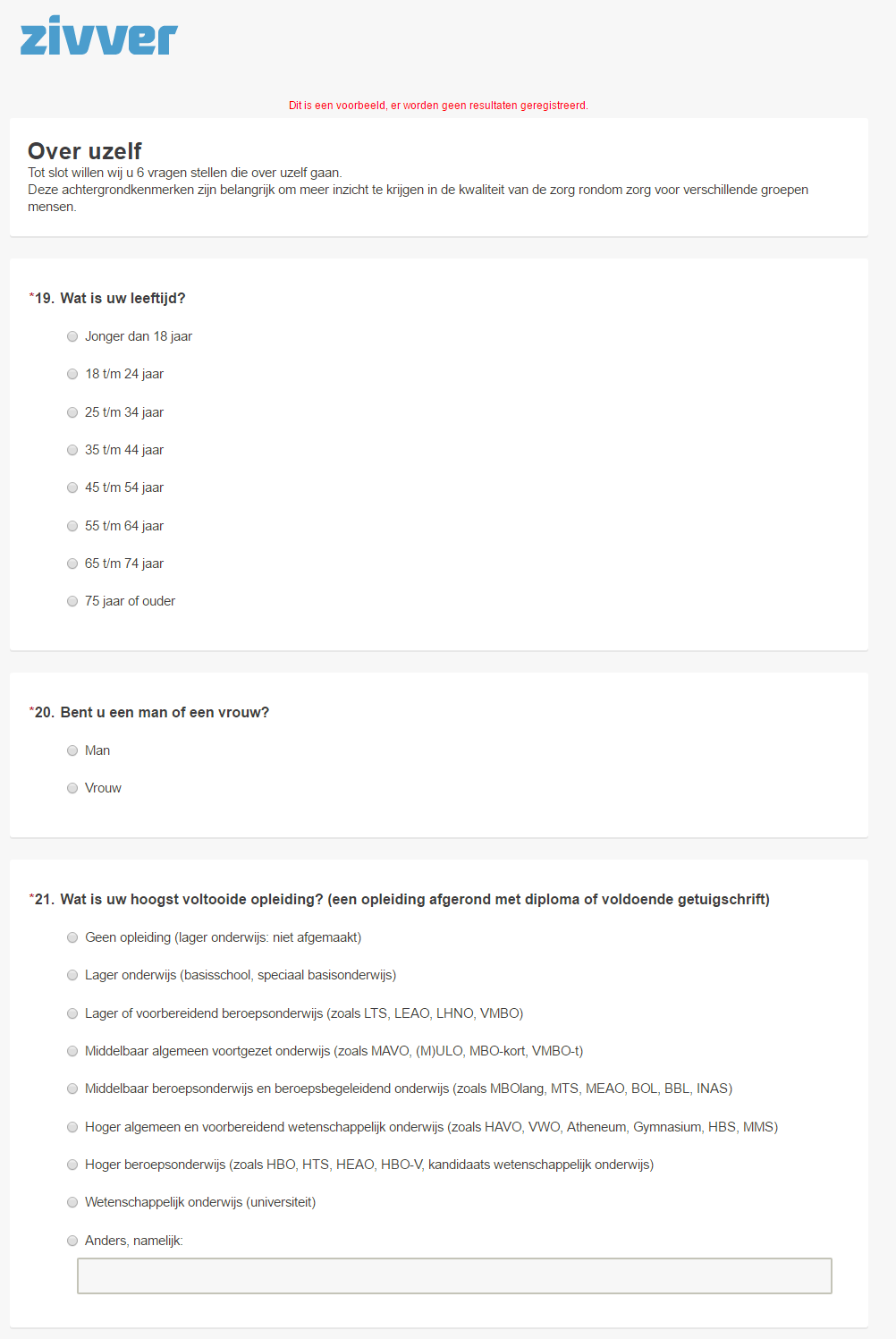

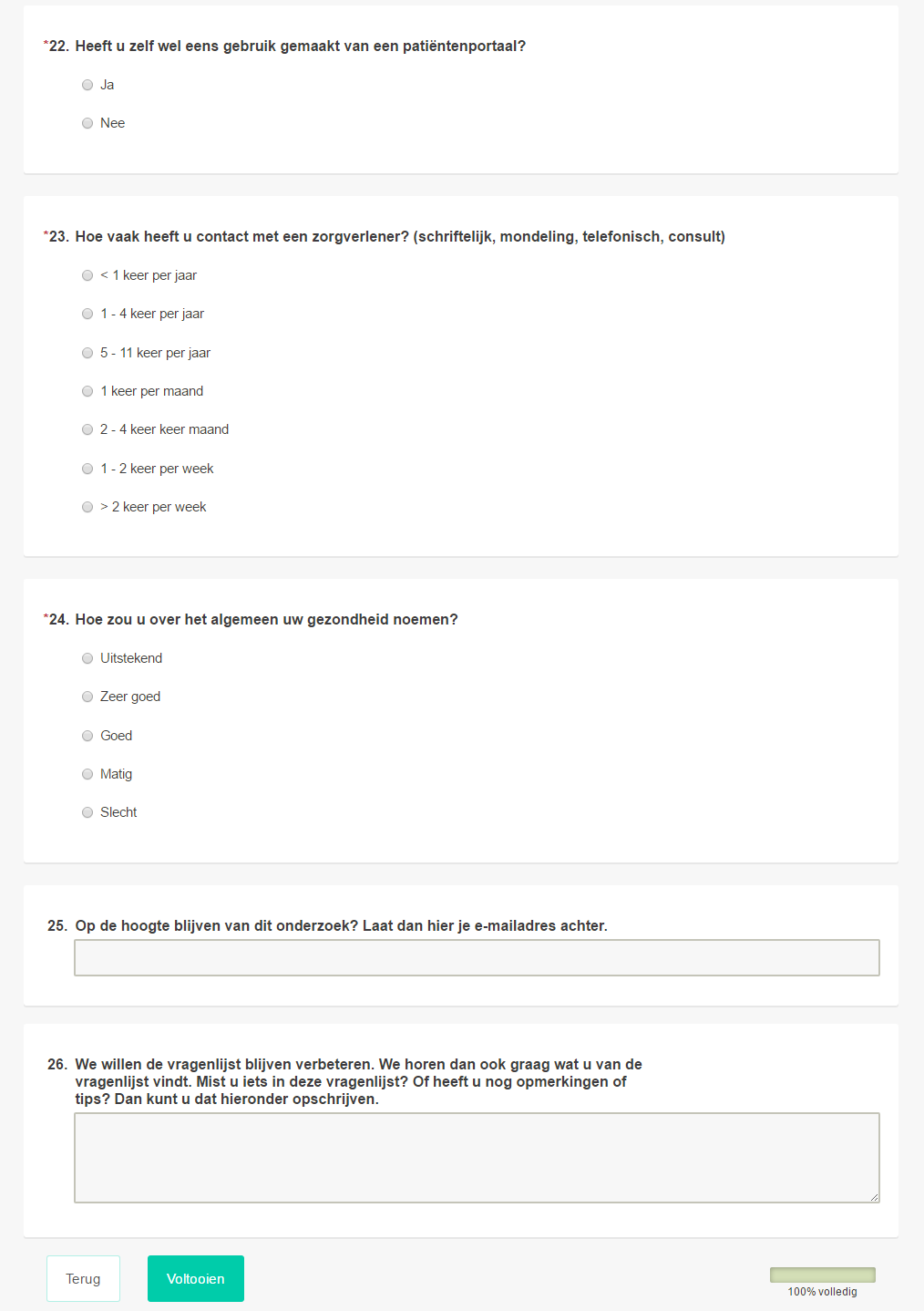


Research Institute Logo 1

Research Institute Logo 1

Research Institute Logo 1

Survey on usage of patient portals

Thank you for participating, completing this survey will only take 10 minutes of your time.

The survey will be used anonymously and confidentially. This means that nobody knows which answers you have given. In addition your personal data will not be shared with others. If you have any questions and/or remarks regarding the survey, you can send an email to [frank.horenberg@zivver.com](mailto:frank.horenberg@zivver.com).

What is a patient portal?

Most questions in this survey will address patient portals. That is why it is important that you understand what a patient portal is. By means of this study your preferences on patient portals are examined, as well as how a patient portal should look like in the future. A patient portal is online environment (for example a website) which you can use to review your own health data. For example, you can use a patient portal to view lab results, such as blood values, or to see your appointments, notes of the doctor or to ask online questions to your healthcare providers.

There are many different patient portals in The Netherlands that are used by your general practitioner or hospitals within your region. Each portal has different functionalities. At some portals you can access your total medical record, at others just some parts of your record. You can watch the short clip (50 seconds) below to learn more on patient portals. If the clip doesn’t load, please click [here](https://www.youtube.com/watch?v=0LmGW4ig2Ys).

What is requested of you?

The previous page explained what is understood by ‘patient portals’. At this page, an example is given on how the survey works. You will get 18 questions on ‘fictive’ portals with 7 different features. Based on these features you will be asked to make a choice between two portals (please see example below). The various portals might not fully comply with your expectations. That is why we ask you to consider with portal option you like most. If both of the options do not comply with your expectations, you can choose ‘none of both’. The text in the grey boxes indicates that that specific feature is similar in portal 1 and 2. An example is shown below:

| Example | | |
| --- | --- | --- |
| Feature | Portal 1 | Portal 2 |
| **Accessibility** | Via computer (i.e. laptop) | Via smartphone (i.e. iPhone) |
| **Login** | Username and password | Username and password with SMS verification |
| **Export** | Data cannot be exported from portal | Data can be put in an overview (i.e. PDF) |
| **Availability** | Information is directly published | Information is directly published |
| **Content** | Test results and basic information; medication overview and allergies | Complete medical record (including possible difficult medical terms) |
| **Aimed at** | Information from one of your health providers (i.e. hospital or general practitioner) | Information from one of your health providers (i.e. hospital or general practitioner) |
| **Interaction** | No possibility to ask online questions | No possibility to ask online questions |
|  | | |

| Which portal would you prefer if you could choose between the portals presented above? |
| --- |
| - Portal 1 |
| - Portal 2 |
| - I will never use portal 1 or portal 2 |

Example of a consideration:

Mister Peters finds it difficult to use a smartphone and downloading information (export) from a portal is not of importance to him. That is why he chooses portal 1 in this example, so he can view is medical information online via his laptop.

In the following 18 questions you will have to make a choice between portal 1 or 2.

The text in the grey boxes indicates that that specific feature is similar in the portals.

**1. Question 1 of 18**

| Feature | | | Portal 1 | Portal 2 | |  |
| --- | --- | --- | --- | --- | --- | --- |
| **Accessibility** | | | Via smartphone (i.e. iPhone) | Via smartphone (i.e. iPhone) | |  |
| **Login** | | | Username and password with SMS verification | Username and password with SMS verification | |  |
| **Export** | | | Data from portal can be exported to other care systems | Data cannot be exported from portal | |  |
| **Availability** | | | Information is published after two weeks, regardless if discussed with health provider | Information is directly published | |  |
| **Content** | | | Complete medical record (including possible difficult medical terms) | Test results and basic information; medication overview and allergies and summaries of consults | |  |
| **Aimed at** | | | Information from one of your health providers (i.e. hospital or general practitioner) | Information from several health providers (i.e. various hospitals and/or general practitioner) | |  |
| **Interaction** | | | Online consult with health provider | Possibility to ask online questions regarding tests and previous appointments | |  |
|  | | Which portal would you prefer if you could choose between the portals presented above? | | |  | |
|  | | - Portal 1 | | |  | |
|  | | - Portal 2 | | |  | |
|  | | - I will never use portal 1 or portal 2 | | |  | |
| *Please note: the question ‘which portal would you prefer if you could choose between the portals presented above?’ was asked at each question from 1 to 18. To show the phrasing of the question, we placed it at question 1 in this translation, yet not at the other boxes.*  **2. Question 2 of 18** | | | | | |  |
| Feature | | | Portal 1 | Portal 2 | |  |
| **Accessibility** | | | Via computer (i.e. laptop) | Via smartphone (i.e. iPhone) | |  |
| **Login** | | | Username and password with SMS verification | Username and password | |  |
| **Export** | | | Data from portal can be exported to other care systems | Data from portal can be exported to other care systems | |  |
| **Availability** | | | Information is published only after being discussed with health provider | Information is published only after being discussed with health provider | |  |
| **Content** | | | Test results and basic information; medication overview and allergies | Test results and basic information; medication overview and allergies and summaries of consults | |  |
| **Aimed at** | | | Information from several health providers (i.e. various hospitals and/or general practitioner) | Information from several health providers (i.e. various hospitals and/or general practitioner) | |  |
| **Interaction** | | | Possibility to ask online questions regarding tests and previous appointments | No possibility to ask online questions | |  |
|  |  | | | |  | |

| **3. Question 3 of 18** | | |
| --- | --- | --- |
| Feature | Portal 1 | Portal 2 |
| **Accessibility** | Via tablet (i.e. iPad) | Via computer (i.e. laptop) |
| **Login** | Username and password with SMS verification | Username and password |
| **Export** | Data cannot be exported from portal | Data can be put in an overview (i.e. PDF) |
| **Availability** | Information is published only after being discussed with health provider | Information is published only after being discussed with health provider |
| **Content** | Complete medical record (including possible difficult medical terms) | Complete medical record (including possible difficult medical terms) |
| **Aimed at** | Information from several health providers (i.e. various hospitals and/or general practitioner) | Information from several health providers (i.e. various hospitals and/or general practitioner) |
| **Interaction** | No possibility to ask online questions | Online consult with health provider |

|  |  |  |
| --- | --- | --- |
|  |  |  |

| **4. Question 4 of 18** | | |
| --- | --- | --- |
| Feature | Portal 1 | Portal 2 |
| **Accessibility** | Via smartphone (i.e. iPhone) | Via tablet (i.e. iPad) |
| **Login** | Username and password | DigiD with SMS verification |
| **Export** | Data from portal can be exported to other care systems | Data can be put in an overview (i.e. PDF) |
| **Availability** | Information is published only after being discussed with health provider | Information is published only after being discussed with health provider |
| **Content** | Test results and basic information; medication overview and allergies and summaries of consults | Test results and basic information; medication overview and allergies and summaries of consults |
| **Aimed at** | Information from one of your health providers (i.e. hospital or general practitioner) | Information from several health providers (i.e. various hospitals and/or general practitioner) |
| **Interaction** | No possibility to ask online questions | Possibility to ask online questions regarding tests and previous appointments |

|  |  |  |
| --- | --- | --- |

|  |
| --- |

| **5. Question 5 of 18** | | |
| --- | --- | --- |
| Feature | Portal 1 | Portal 2 |
| **Accessibility** | Via computer (i.e. laptop) | Via smartphone (i.e. iPhone) |
| **Login** | DigiD with SMS verification | Username and password with SMS verification |
| **Export** | Data from portal can be exported to other care systems | Data from portal can be exported to other care systems |
| **Availability** | Information is directly published | Information is published after two weeks, regardless if discussed with health provider |
| **Content** | Test results and basic information; medication overview and allergies and summaries of consults | Complete medical record (including possible difficult medical terms) |
| **Aimed at** | Information from several health providers (i.e. various hospitals and/or general practitioner) | Information from several health providers (i.e. various hospitals and/or general practitioner) |
| **Interaction** | Online consult with health provider | Online consult with health provider |

| **6. Question 6 of 18** | | |
| --- | --- | --- |
| Feature | Portal 1 | Portal 2 |
| **Accessibility** | Via smartphone (i.e. iPhone) | Via computer (i.e. laptop) |
| **Login** | Username and password with SMS verification | Username and password with SMS verification |
| **Export** | Data cannot be exported from portal | Data from portal can be exported to other care systems |
| **Availability** | Information is directly published | Information is published only after being discussed with health provider |
| **Content** | Test results and basic information; medication overview and allergies and summaries of consults | Test results and basic information; medication overview and allergies |
| **Aimed at** | Information from one of your health providers (i.e. hospital or general practitioner) | Information from one of your health providers (i.e. hospital or general practitioner) |
| **Interaction** | Possibility to ask online questions regarding tests and previous appointments | Possibility to ask online questions regarding tests and previous appointments |

| **7. Question 7 of 18** | | |
| --- | --- | --- |
| Feature | Portal 1 | Portal 2 |
| **Accessibility** | Via computer (i.e. laptop) | Via smartphone (i.e. iPhone) |
| **Login** | Username and password | DigiD with SMS verification |
| **Export** | Data cannot be exported from portal | Data can be put in an overview (i.e. PDF) |
| **Availability** | Information is directly published | Information is directly published |
| **Content** | Test results and basic information; medication overview and allergies | Complete medical record (including possible difficult medical terms) |
| **Aimed at** | Information from one of your health providers (i.e. hospital or general practitioner) | Information from one of your health providers (i.e. hospital or general practitioner) |
| **Interaction** | No possibility to ask online questions | No possibility to ask online questions |

| **8. Question 8 of 18** | | |
| --- | --- | --- |
| Feature | Portal 1 | Portal 2 |
| **Accessibility** | Via smartphone (i.e. iPhone) | Via computer (i.e. laptop) |
| **Login** | Username and password | DigiD with SMS verification |
| **Export** | Data can be put in an overview (i.e. PDF) | Data cannot be exported from portal |
| **Availability** | Information is published after two weeks, regardless if discussed with health provider | Information is published after two weeks, regardless if discussed with health provider |
| **Content** | Test results and basic information; medication overview and allergies | Complete medical record (including possible difficult medical terms) |
| **Aimed at** | Information from several health providers (i.e. various hospitals and/or general practitioner) | Information from several health providers (i.e. various hospitals and/or general practitioner) |
| **Interaction** | Possibility to ask online questions regarding tests and previous appointments | Possibility to ask online questions regarding tests and previous appointments |

|  |  |
| --- | --- |

| **10. Question 10 of 18** | | |
| --- | --- | --- |
| Feature | Portal 1 | Portal 2 |
| **Accessibility** | Via tablet (i.e. iPad) | Via computer (i.e. laptop) |
| **Login** | Username and password | Username and password with SMS verification |
| **Export** | Data cannot be exported from portal | Data can be put in an overview (i.e. PDF) |
| **Availability** | Information is published after two weeks, regardless if discussed with health provider | Information is published after two weeks, regardless if discussed with health provider |
| **Content** | Test results and basic information; medication overview and allergies and summaries of consults | Test results and basic information; medication overview and allergies and summaries of consults |
| **Aimed at** | Information from several health providers (i.e. various hospitals and/or general practitioner) | Information from one of your health providers (i.e. hospital or general practitioner) |
| **Interaction** | Online consult with health provider | No possibility to ask online questions |

| **9. Question 9 of 18** | | |
| --- | --- | --- |
| Feature | Portal 1 | Portal 2 |
| **Accessibility** | Via tablet (i.e. iPad) | Via tablet (i.e. iPad) |
| **Login** | Username and password | DigiD with SMS verification |
| **Export** | Data from portal can be exported to other care systems | Data from portal can be exported to other care systems |
| **Availability** | Information is directly published | Information is published after two weeks, regardless if discussed with health provider |
| **Content** | Complete medical record (including possible difficult medical terms) | Test results and basic information; medication overview and allergies |
| **Aimed at** | Information from several health providers (i.e. various hospitals and/or general practitioner) | Information from several health providers (i.e. various hospitals and/or general practitioner) |
| **Interaction** | Possibility to ask online questions regarding tests and previous appointments | No possibility to ask online questions |

| **11.. Question 11 off 18** | | |
| --- | --- | --- |
| Feature | Portal 1 | Portal 2 |
| **Accessibility** | Via computer (i.e. laptop) | Via computer (i.e. laptop) |
| **Login** | Username and password | Username and password |
| **Export** | Data can be put in an overview (i.e. PDF) | Data cannot be exported from portal |
| **Availability** | Information is published only after being discussed with health provider | Information is directly published |
| **Content** | Complete medical record (including possible difficult medical terms) | Test results and basic information; medication overview and allergies |
| **Aimed at** | Information from one of your health providers (i.e. hospital or general practitioner) | Information from several health providers (i.e. various hospitals and/or general practitioner) |
| **Interaction** | Online consult with health provider | No possibility to ask online questions |

| **12. Question 12 of 18** | | |
| --- | --- | --- |
| Feature | Portal 1 | Portal 2 |
| **Accessibility** | Via smartphone (i.e. iPhone) | Via tablet (i.e. iPad) |
| **Login** | DigiD with SMS verification | Username and password with SMS verification |
| **Export** | Data can be put in an overview (i.e. PDF) | Data can be put in an overview (i.e. PDF) |
| **Availability** | Information is directly published | Information is directly published |
| **Content** | Complete medical record (including possible difficult medical terms) | Test results and basic information; medication overview and allergies |
| **Aimed at** | Information from several health providers (i.e. various hospitals and/or general practitioner) | Information from several health providers (i.e. various hospitals and/or general practitioner) |
| **Interaction** | No possibility to ask online questions | Online consult with health provider |

| **13. Question 13 of 18** | | |
| --- | --- | --- |
| Feature | Portal 1 | Portal 2 |
| **Accessibility** | Via tablet (i.e. iPad) | Via computer (i.e. laptop) |
| **Login** | Username and password with SMS verification | DigiD with SMS verification |
| **Export** | Data can be put in an overview (i.e. PDF) | Data from portal can be exported to other care systems |
| **Availability** | Information is directly published | Information is directly published |
| **Content** | Test results and basic information; medication overview and allergies | Test results and basic information; medication overview and allergies and summaries of consults |
| **Aimed at** | Information from one of your health providers (i.e. hospital or general practitioner) | Information from one of your health providers (i.e. hospital or general practitioner) |
| **Interaction** | Online consult with health provider | Online consult with health provider |

| **14. Question 14 of 18** | | |
| --- | --- | --- |
| Feature | Portal 1 | Portal 2 |
| **Accessibility** | Via smartphone (i.e. iPhone) | Via tablet (i.e. iPad) |
| **Login** | DigiD with SMS verification | Username and password with SMS verification |
| **Export** | Data cannot be exported from portal | Data cannot be exported from portal |
| **Availability** | Information is published only after being discussed with health provider | Information is published only after being discussed with health provider |
| **Content** | Test results and basic information; medication overview and allergies | Complete medical record (including possible difficult medical terms) |
| **Aimed at** | Information from several health providers (i.e. various hospitals and/or general practitioner) | Information from one of your health providers (i.e. hospital or general practitioner) |
| **Interaction** | Online consult with health provider | No possibility to ask online questions |

| **15. Question 15 of 18** | | |
| --- | --- | --- |
| Feature | Portal 1 | Portal 2 |
| **Accessibility** | Via computer (i.e. laptop) | Via tablet (i.e. iPad) |
| **Login** | Username and password with SMS verification | Username and password |
| **Export** | Data can be put in an overview (i.e. PDF) | Data from portal can be exported to other care systems |
| **Availability** | Information is published after two weeks, regardless if discussed with health provider | Information is directly published |
| **Content** | Test results and basic information; medication overview and allergies and summaries of consults | Complete medical record (including possible difficult medical terms) |
| **Aimed at** | Information from several health providers (i.e. various hospitals and/or general practitioner) | Information from one of your health providers (i.e. hospital or general practitioner) |
| **Interaction** | No possibility to ask online questions | Possibility to ask online questions regarding tests and previous appointments |

| **16. Question 16 of 18** | | |
| --- | --- | --- |
| Feature | Portal 1 | Portal 2 |
| **Accessibility** | Via computer (i.e. laptop) | Via tablet (i.e. iPad) |
| **Login** | DigiD with SMS verification | Username and password |
| **Export** | Data cannot be exported from portal | Data cannot be exported from portal |
| **Availability** | Information is published after two weeks, regardless if discussed with health provider | Information is published after two weeks, regardless if discussed with health provider |
| **Content** | Complete medical record (including possible difficult medical terms) | Test results and basic information; medication overview and allergies and summaries of consults |
| **Aimed at** | Information from one of your health providers (i.e. hospital or general practitioner) | Information from one of your health providers (i.e. hospital or general practitioner) |
| **Interaction** | Possibility to ask online questions regarding tests and previous appointments | Online consult with health provider |

| **17. Question 17 of 18** | | |
| --- | --- | --- |
| Feature | Portal 1 | Portal 2 |
| **Accessibility** | Via tablet (i.e. iPad) | Via smartphone (i.e. iPhone) |
| **Login** | DigiD with SMS verification | DigiD with SMS verification |
| **Export** | Data can be put in an overview (i.e. PDF) | Data cannot be exported from portal |
| **Availability** | Information is published only after being discussed with health provider | Information is published only after being discussed with health provider |
| **Content** | Test results and basic information; medication overview and allergies and summaries of consults | Test results and basic information; medication overview and allergies |
| **Aimed at** | Information from one of your health providers (i.e. hospital or general practitioner) | Information from one of your health providers (i.e. hospital or general practitioner) |
| **Interaction** | Possibility to ask online questions regarding tests and previous appointments | Online consult with health provider |

| **18. Question 18 of 18** | | |
| --- | --- | --- |
| Feature | Portal 1 | Portal 2 |
| **Accessibility** | Via tablet (i.e. iPad) | Via smartphone (i.e. iPhone) |
| **Login** | DigiD with SMS verification | Username and password |
| **Export** | Data from portal can be exported to other care systems | Data can be put in an overview (i.e. PDF) |
| **Availability** | Information is published after two weeks, regardless if discussed with health provider | Information is published after two weeks, regardless if discussed with health provider |
| **Content** | Test results and basic information; medication overview and allergies | Test results and basic information; medication overview and allergies |
| **Aimed at** | Information from one of your health providers (i.e. hospital or general practitioner) | Information from one of your health providers (i.e. hospital or general practitioner) |
| **Interaction** | No possibility to ask online questions | Possibility to ask online questions regarding tests and previous appointments |

Regarding yourself.

To conclude we would like to ask you 6 questions regarding yourself. These characteristics are important to get more sight on the quality of care for different groups of people.

| * | 19. What is your age? |  |
| --- | --- | --- |
|  | - Younger than 18 years |  |
|  | - 18 till 24 years |  |
|  | - 25 till 34 years |  |
|  | - 35 till 44 years |  |
|  | - 45 till 54 years |  |
|  | - 55 till 64 years |  |
|  | - 65 till 74 years |  |
|  | - 75 years and above |  |
|  |  |  |

| * | 20. Are you male or female? |  |
| --- | --- | --- |
|  | - Male |  |
|  | - Female |  |
|  |  |  |

| * | 21. What is your highest completed education? (Education completed with a degree or a certificate) |  |
| --- | --- | --- |
|  | - No education (primary school: not finished) |  |
|  | - Lower education (primary school, special primary school) |  |
|  | - Lower or preparatory vocational education (such as LTS, LEAO, LHNO, VMBO) |  |
|  | - Middle-level secundary education (such as MAVO, (M)ULO, MBO-kort, VMBO-t) |  |
|  | - Middle-level vocation education (such as MBOlang, MTS, MEAO, BOL, BBL, INAS) |  |
|  | - Higher secundary education (such as HAVO, VWO, Atheneum, Gymnasium, HBS, MMS) |  |
|  | - Higher vocation education (such as HBO, HTS, HEAO, HBO-V, candidates scientific education) |  |
|  | - Scientific education (university) |  |
|  | - Other, please specify:  ............................................................ |  |
|  |  |  |

| * | 22. Have you ever used a patient portal yourself? |  |
| --- | --- | --- |
|  | - Yes |  |
|  | - No |  |
|  |  |  |

| * | 23. How often are you in contact with a healthcare provider? (written, face-to-face, by telephone, via a consult) |  |
| --- | --- | --- |
|  | - < 1 time per year |  |
|  | - 1 - 4 times per year |  |
|  | - 5 - 11 times per year |  |
|  | - 1 time per month |  |
|  | - 2 - 4 times per month |  |
|  | - 1 - 2 times per week |  |
|  | - > 2 times per week |  |
|  |  |  |

| * | 24. In general, how do your describe your health? |  |
| --- | --- | --- |
|  | - Excellent |  |
|  | - Very good |  |
|  | - Good |  |
|  | - Poor |  |
|  | - Bad |  |
|  |  |  |

|  | 25. Would you like to be updated on this research? If so, please register your email address. |  |
| --- | --- | --- |

|  | 26. We would like to improve the survey. For this reason we would like to hear your opinion on the survey. Was something lacking in the survey? Do you have any remarks or tips regarding the survey? You can give your feedback in the box below. |  |
| --- | --- | --- |
|  |  |  |
|  |  |  |
